# Design, Synthesis, and Biological Evaluation of 4,4’-Difluorobenzhydrol Carbamates as Selective M_1_ Antagonists

**DOI:** 10.3390/ph15020248

**Published:** 2022-02-18

**Authors:** Jonas Kilian, Marius Ozenil, Marlon Millard, Dorka Fürtös, Verena Maisetschläger, Wolfgang Holzer, Wolfgang Wadsak, Marcus Hacker, Thierry Langer, Verena Pichler

**Affiliations:** 1 Department of Biomedical Imaging and Image-Guided Therapy, Division of Nuclear Medicine, Medical University of Vienna, 1090 Vienna, Austria; jonas.kilian@meduniwien.ac.at (J.K.); marius.ozenil@meduniwien.ac.at (M.O.); wolfgang.wadsak@meduniwien.ac.at (W.W.); marcus.hacker@meduniwien.ac.at (M.H.); 2 Department of Pharmaceutical Sciences, Division of Pharmaceutical Chemistry, Faculty of Life Sciences, University of Vienna, 1090 Vienna, Austria; marlon.millard@univie.ac.at (M.M.); a01546417@unet.univie.ac.at (D.F.); a01631177@unet.univie.ac.at (V.M.); wolfgang.holzer@univie.ac.at (W.H.); thierry.langer@univie.ac.at (T.L.); 3 CBmed GmbH—Center for Biomarker Research in Medicine, 8036 Graz, Austria

**Keywords:** muscarinic acetylcholine receptors, subtype selectivity, drug development, molecular docking

## Abstract

Due to their important role in mediating a broad range of physiological functions, muscarinic acetylcholine receptors (mAChRs) have been a promising target for therapeutic and diagnostic applications alike; however, the list of truly subtype-selective ligands is scarce. Within this work, we have identified a series of twelve 4,4’-difluorobenzhydrol carbamates through a rigorous docking campaign leveraging commercially available amine databases. After synthesis, these compounds have been evaluated for their physico–chemical property profiles, including characteristics such as HPLC-logD, tPSA, logBB, and logPS. For all the synthesized carbamates, these characteristics indicate the potential for BBB permeation. In competitive radioligand binding experiments using Chinese hamster ovary cell membranes expressing the individual human mAChR subtype *h*M_1_-*h*M_5_, the most promising compound 2 displayed a high binding affinitiy towards *h*M_1_R (1.2 nM) while exhibiting modest-to-excellent selectivity versus the *h*M_2-5_R (4–189-fold). All 12 compounds were shown to act in an antagonistic fashion towards *h*M1R using a dose-dependent calcium mobilization assay. The structural eligibility for radiolabeling and their pharmacological and physico–chemical property profiles render compounds 2, 5, and 7 promising candidates for future position emission tomography (PET) tracer development.

## 1. Introduction

**Figure 1:**
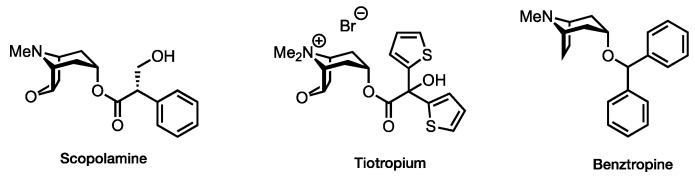
Examples of clinically approved pan-antimuscarinic drugs.

Muscarinic acetylcholine receptors (mAChRs) belong to the superfamily of G-protein-coupled receptors (GPCRs) which, upon activation by their endogenous neurotransmitter acetylcholine, elicit a multitude of peripheral and central physiological functions such as cognitive function, motor control, and cardiovascular function. There are five subtypes of mAChRs (M_1_–M_5_), all of which are expressed in varying degrees throughout the human body [1]. Their abundant expression in the central nervous system (CNS), in particular, led to them being the therapeutic target of numerous research efforts targeting pathologies such as Alzheimer’s disease, Parkinson’s disease, and schizophrenia [2,3,4,5]; however, these efforts have not been the most fruitful—attributable to the highly conserved orthosteric binding site shared among M_1_–M_5_, posing a severe constraint on subtype-selective drug development [2]. Not only is the design of ligands preferably targeting, for example, the M_1_ or M_4_ receptors, known targets for neurological diseases [6,7], a difficult task to achieve, but non-selective compounds suffer from dose-limiting adverse effects [8]. These commonly spring from the unwanted activation of peripheral M_2_ and M_3_ receptors [7]. As a result, clinicians’ shelves are characterized by a lack of truly subtype-selective mAChR ligands. Instead, a range of side effect-plagued pan-muscarinic antagonists and inverse agonists is used in clinical practice, such as the antiemetic agent scopolamine, the bronchodilator tiotropium, or benztropine which is used to treat symptoms of Parkinson’s disease (Figure 1). Thus, current research is increasingly devoted towards the discovery of more selective ligands targeting an allosteric site exhibiting less sequence homology or so-called bitopic ligands, simultaneously targeting the orthosteric and an allosteric site [9,10].

The abundant expression of mAChRs in brain tissue also renders them a promising target in CNS-targeting positron emission tomography (PET) applications, a non-invasive imaging technique offering a wide range of functional information such as quantifying the distribution, expression, and modulation of the targeted receptor in normal and pathologically changed tissue [11]. As such, a PET tracer targeting individual mAChR subtypes could contribute immensely to the understanding of muscarinic receptor signaling in brain physiology, and its role in neurological pathophysiology. 

As evidence for the promising role of selective M_1_ targeting antagonists in the treatment of many neurological indications including Parkinson’s disease and multiple sclerosis accumulates [12,13], we sought to identify an M_1_ muscarinic ligand, displaying a suitable selectivity profile versus M_2_–M_5_ paired with a sufficiently high affinity (approx. 3–50 nM) for a potential application as a PET imaging probe [14]. Such a probe in turn could, for example, facilitate compound selection for clinical trials by providing in vivo occupancy data [15].

**Figure 2:**
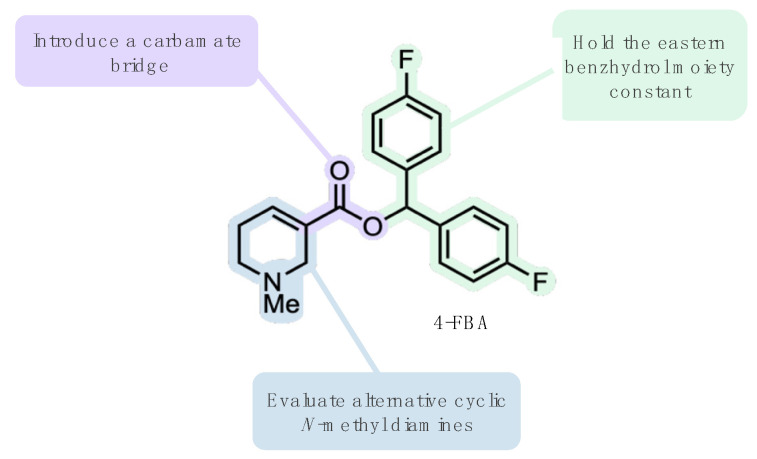
General structural modification strategy of parent compound 4-FBA [16].

Our group recently made tangible progress in this direction with the discovery of highly M_1_ selective benzhydrol esters of arecaidine with *K*_i_ values in the single-digit nanomolar range [16]; however, excessive non-displaceable binding (NDB) limits the usability of these ligands for molecular imaging purposes [17]. Thus, in this study, we envisioned structural modifications of the bis(4-fluorophenyl)methyl 1-methyl-1,2,5,6-tetrahydropyridine-3-carboxylate scaffold (4-FBA, Figure 2), which may result in lower non-displaceable binding while retaining the favorable binding properties. Herein, we report a docking campaign, the synthesis, and physico–chemical and pharmacological evaluation of a new series of 4,4’-difluorobenzhydrol carbamates acting as ligands of the M_1_ muscarinic receptor.

## 2. Results and Discussion

### 2.1. Ligand Design

To assess whether the envisioned structural modification of 4-FBA will lead to suitable muscarinic ligands, we undertook a docking campaign against the M_1_ muscarinic receptor structure (PDB 5CXV). This crystal structure of the inactive M_1_ receptor features a, within the transmembrane core, deeply buried orthosteric binding pocket occupied by its co-crystallized small molecule inverse agonist tiotropium [18]. Within the binding site, tiotropium’s spatial orientation is such that it simultaneously fills two lipophilic pockets with its thiophene rings, while opposite to this region its carbonyl oxygen and its hydroxyl group act as hydrogen bond acceptor and donor towards Asn382^6.52^, respectively (superscript numerals refer to the Ballesteros-Weinstein numbering scheme for GPCRs [19]). Additionally, the cationic amine forms a salt bridge with Asp105^3.32^, a residue which is conserved among other aminergic GPCRs [20]. 

**Figure 3:**
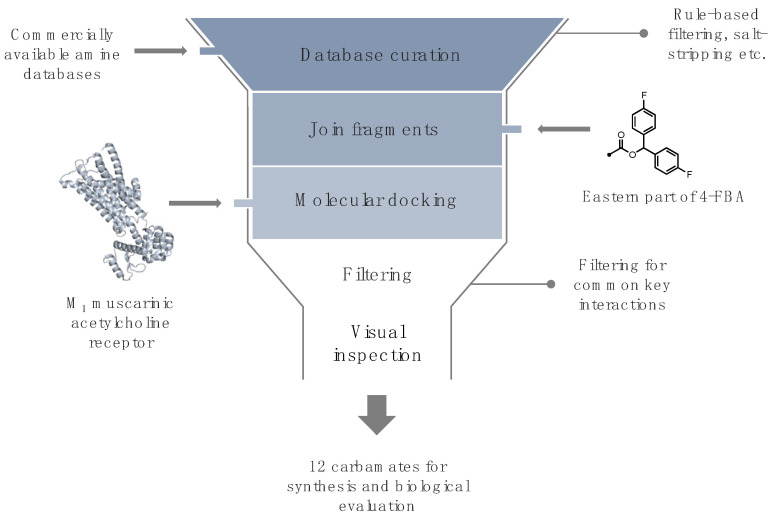
Schematic depiction of the utilized in silico workflow in this study.

We started our in silico (Figure 3) workflow to design and evaluate carbamate-bridged compounds based on 4-FBA by preparing a narrowly focused library of commercially available diamines, with the structural prerequisite of one amine moiety being an aliphatic tertiary *N*-methyl amine enclosed in a cyclic structure. The rationale for this was three-fold: firstly, structural rigidification of the amine part of the molecule may lead to an increase in binding affinity by limiting the rotational freedom of this group. Secondly, due to the basicity of this structural element, the amine should, under physiological conditions, exist at least partly in its protonated form, thereby enabling the possibility of an ionic interaction with Asp105^3.32^ similar to tiotropium. Thirdly, considering the potential application as PET imaging probes, such compounds, contrasting cyclic tertiary amine structures such as quinuclidine, would be amenable to straightforward radiolabeling with carbon-11. More precisely, merging in-stock primary and secondary amines from Enamine with in-stock diamines from Chemspace led to the creation of a compound library counting 52,857 amines. After curating this library by, for example, salt-stripping and dropping duplicates, and applying the above elucidated filter criterion, undefined stereocenters were enumerated, resulting in a dramatically reduced selection of 331 diamine fragments. This selection was subsequently linked with 4-FBA’s eastern 4,4’-difluorobenzhydrol via a carbamate bridge and the resulting carbamates were set to their energetically most favorable ionization state at pH 7.4 and subjected to docking. To prioritize among the docked compounds, a distance filter has been used, dropping all poses whose cationic amine did not come within a distance of 5.5 Å to the Asp105^3.32^ carboxyl oxygens. Since the charge-charge interaction between Asp105^3.32^ and a ligand’s cationic head is not restricted to a distinct spatial point, the ammonium group’s position has some leeway [10]. The distance of tiotropium’s positively charged amine to the Asp105^3.32^ carboxyl oxygens is slightly below 5 Å, and for other known ligands, the distance is predicted to be in a similar range [18,21], hence a distance constraint of 5.5 Å was assumed to be reasonable. For each ligand–receptor complex, only the top ranked pose exhibiting an ionic interaction with Asp105^3.32^ according to LigandScout was selected as a representative [22], leaving a final dataset of 129 potential ligands. Considering the approximate nature of docking scores and the corresponding interaction energies, compound ranking based on these metrices is insufficient [23]; instead, we visually inspected the remaining compounds and selected a set of 12 that interacted with similar residues as those predicted for 4-FBA, such as Cys407^7.42^, Tyr106^3.33^ and Thr189^5.39^ for further experimental validation [16].

**Figure 4:**
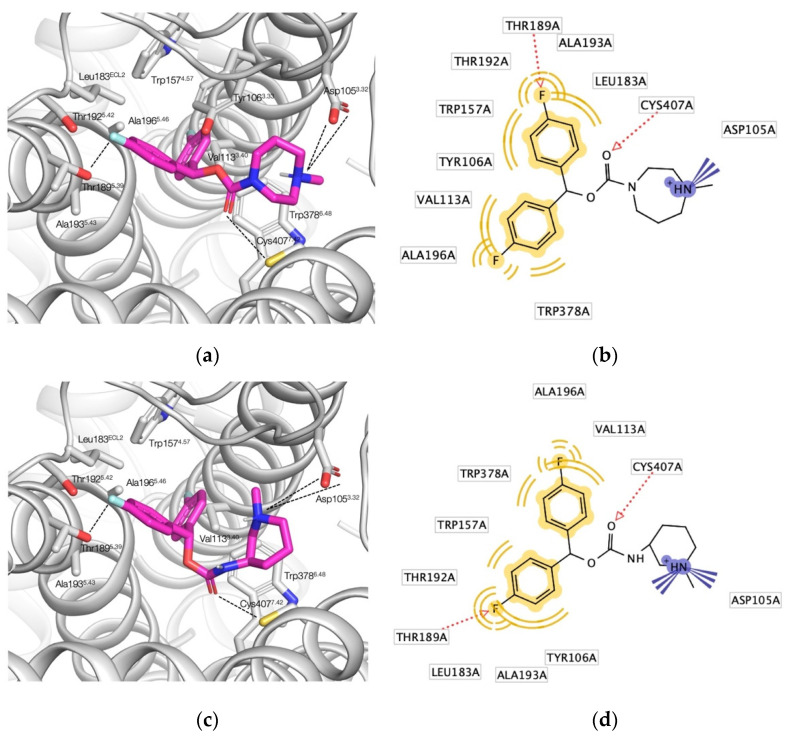
Docking poses for selected hits (carbons in magenta) in the orthosteric binding site of M_1_ (PDB 5CXV) with interacting amino acid residues and key polar interactions highlighted (dashed lines) and the corresponding 2D pharmacophores: (**a**) docking pose of **2**; (**b**) 2D pharmacophore of **2**; (**c**) docking pose of (*R*)-**7**; Tyr106^3.33^ and Tyr404^7.39^ omitted for the sake of clarity; (**d**) 2D pharmacophore of (*R*)-**7**.

Overall, the selected carbamates **1**–**12** engage in similar pharmacophoric interactions with the orthosteric binding site of the M_1_ muscarinic receptor. Compound **2** adopts an extended pose, engaging with many of the residues featured in the binding mode of tiotropium (Figure 4a,b) [18]. As required by our post-docking filter, the 1,4-diazepane’s *N*-methyl ammonium group forms a salt bridge with Asp105^3.32^; however, unlike many known agonists and antagonists, **2** does not interact with Asn382^6.52^ [24], instead it is predicted to form a hydrogen bond with Cys407^7.42^. In fact, this interaction with Cys407^7.42^ together with a fluorine–hydrogen bond with Thr189^5.39^ is shared among the whole compound selection except for **5** (Supplementary Materials, Figure S1). The carbonyl oxygen of spirocyclic **5**, by contrast, acts as a hydrogen bond acceptor for Tyr106^3.33^. Compounds **3**, **4**, and **8** not only engage in hydrogen bonding with Cys407^7.42^, but form yet another hydrogen bond with Asn382^6.52^ (Supplementary Materials, Figure S1d,f,l). While the fluorinated benzhydrol part of the molecules **2** and (*R*)-**7** adopt an almost identical pose, occupying the binding site’s two lipophilic pockets, the ammonium groups differ slightly in their spatial position attributable to the different ring geometries (Figure 4c). Compared to **2**, the protonated amine of (*R*)-**7**’s piperidine ring is tilted, enabling another favorable cation–π interaction with Tyr404^7.39^, a key interaction in many known muscarinic ligands (Figure 4d) [21]. The enantiomeric pairs of the secondary carbamates **7** and **9**–**12** are predicted to adopt largely overlapping poses (Supplementary Materials, Figure S2). Since it was difficult to identify any stereospecificity of the pharmacophore from those, we decided to move along with the racemic versions of the aforementioned compounds at this stage, having the additional benefit of speeding up the biological testing.

### 2.2. Chemistry

The synthetic route towards the 4,4’-difluorobenzhydrol carbamate derivatives **1**–**12** is outlined in Table 1. Briefly, treating commercially available primary or secondary amines attached to an aliphatic tertiary *N*-methyl amine enclosed in a cyclic structure with *N,N*’-carbonyldiimidazole (CDI) in DMF at ambient temperature gave an intermediary carbamoylimidazole. Subsequent treatment with the sodium alkoxide derived from 4,4’-difluorinated benzhydrol gave the desired carbamate-bridged compounds **1**–**12** in moderate yields ranging from 10 to 38%. 

Considering the presence of a carbamate motif and its somewhat restricted C–N bond rotation, it is unsurprisingly that for many of the synthesized compounds, two rotameric species have been observed in ^1^H and/or ^13^C NMR spectra. For the tertiary carbamates **3** and **5,** two complete sets of signals for the *syn*- and *anti*-rotamers have been detected. In both of the latter cases, the ratio between the rotameric species is 1.25:1. This ratio is in accordance with the rather low energy barrier to C–N bond rotation found in carbamates, often resulting in rotameric ratios close to 1:1 [25]. Other compounds, such as the secondary carbamates **11** and **12** merely show a partial splitting of some aromatic signals in the corresponding ^13^C NMR spectra. 

### 2.3. Physico–Chemical Property Profile and Stability

Bearing the potential application of the designed carbamates as central nervous system PET tracers in mind, we opted to evaluate selected physico–chemical properties, such as lipophilicity, serving as approximate surrogate measures of NDB and blood–brain barrier (BBB) permeability. Although the predictive power of, for example, logP or logD, for NDB or BBB penetration is critically debated, their influence on the aforementioned phenomena is undisputed [26,27,28,29]. 

The lipophilicity of **1**–**12** was estimated by HPLC-logD, a high throughput chromatographic method employing an octadecyl-poly(vinyl alcohol) stationary phase [27,30]. Considering the moderately strong basicity of the analyzed carbamates due to the presence of a tertiary *N*-methyl amine (Table 2), logD at pH 7.4 is preferable to logP since it factors in p*K*_a_. Overall, the HPLC-logD values of the synthesized carbamates were found to be in a narrow range of 2.2–3.25 (Table 2). Notably, the calculated logD values are in satisfactory accordance with the measured HPLC-logD values; only in case of more basic carbamates, the values diverge for some compounds, e.g., **10** and **11**. Compared to the recently published highly M_1_-selective 4-FBA [16], the lipophilicity of this set of compounds is lower, enabling the assumption of lower NDB. Furthermore, all measured HPLC-logD values as well as the calculated tPSA (total polar surface area) values are in the range of established BBB permeable radiotracers (logD: 1–5; tPSA: <90 Å^2^) [31], supporting BBB penetration. If, however, one consults a different logD guideline (1.2–3.1) for centrally acting drugs [32], carbamates **1**, **2**, **3**, **6**, and **8** can be coined borderline cases. The calculated p*K*_a_ values are all within the suggested range (<10.5) [33], with the exception of **8** (10.9).

Other molecular descriptors which are commonly used in in silico models to predict BBB penetration are logBB and logPS [35,36]. While logBB is a logarithmic expression for the equilibrium ratio of the concentration of a compound in brain to that in plasma [31], logPS is a measure for the rate of brain penetration [36]. Similar to logD, the significance of both molecular descriptors is controversially discussed [37,38,39,40,41]; however, since these descriptors represent only a part of our physico–chemical property analysis, their use has been deemed appropriate. 

Thresholds that have been reported in the literature, corresponding to BBB permeability, are logBB > 0.3 and logPS > −2 [31,36]. Hence, these calculated descriptors further strengthen the assumption of BBB permeability for carbamates **1**–**11** (Table 2). Compound **12,** on the other hand, according to its logBB value (0.23), is predicted to be BBB impermeable.

Since the decomposition in cell culture medium of compounds designated for biological testing could impair potential assay readouts, the stabilities of one tertiary carbamate (**3**) and one secondary carbamate (**7**) were investigated as representatives for the compound set **1**–**12**. Gratifyingly, the rate of decomposition in fully supplemented RPMI1640 cell culture medium at ambient temperature has been fairly slow, with > 95% of both compounds remaining intact after 24 h (Supplementary Materials, Figure S3). Such stability is adequate with the requirements for carbon-11 labelled PET tracers of around 2–3 half-lives.

### 2.4. Biological Evaluation

To rule out any distortion of further affinity and functionality testing, cell viability of **1**–**12** was assessed in living CHO-*h*M_1_ cells using an MTT assay and found to be unaffected in the concentration ranges of interest to us, with IC_50_ values corresponding to cytotoxicity in the double and triple digit micromolar range (Supplementary Materials, Figure S4).

We first assessed the carbamates’ affinities for human muscarinic acetylcholine receptors subtypes *h*M_1–5_ by means of a competitive radioligand binding assay displacing [*N*-methyl-^3^H]scopolamine methyl chloride ([^3^H]NMS) in cell membranes expressing the individual receptors. To streamline the time-consuming and expensive process of affinity testing, preliminary single-concentration displacement assays were performed for all compounds at ligand concentrations corresponding to a *K*_i_ value of 1 µM according to the Cheng–Prusoff Equation. As only those compounds with a *K*_i_ in the low nanomolar range will be of importance for PET tracer development, only compounds exhibiting greater than 70% radioligand displacement at any of the subtypes were subsequently exhaustively profiled against the complete set of *h*M_1-5_ receptors in concentration-dependent displacement assays to determine their inhibition constants (*K*_i_). While none of the tested compounds was devoid of any affinity for mAChRs in the preliminary screening experiments, the secondary carbamates **6**, **11**, and **12** did not qualify for further evaluations (Supplementary Materials, Table S1). 

Of all tested compounds, the 1-methyl-1,4-diazepane containing tertiary carbamate **2** and the 1-methylpiperidin-3-amine containing secondary carbamate **7** displayed the highest affinity towards *h*M_1_R with almost equal *K*_i_ values of 1.2 nM and 1.22 nM, respectively (Table 3). Interestingly, both compounds follow the same selectivity trend, i.e., decreasing affinities in the order *h*M_1_R > *h*M_5_R > *h*M_4_R > *h*M_3_R > *h*M_2_R; however, while **7** shows moderate *h*M_1_ selectivity over the *h*M_2-5_ subtypes, **2** exhibits good-to-excellent selectivity versus the *h*M_2-4_R (up to 189-fold) with a slightly lower 4-fold selectivity versus the *h*M_5_R. This pharmacological profile markedly outperforms this study’s parent molecule 4-FBA and our recently published hydrobenzoin esters of arecaidine selectivity-wise [16,42], thereby rendering **2** our group’s most promising *h*M_1_ preferring candidate in terms of subtype selectivity to date. With the exception of **8**, all tested compounds are *h*M_1_ preferring and display by far their highest selectivity against *h*M_2_R, ranging from 30-fold to 189-fold for the tertiary carbamates **1**–**5**. The secondary carbamates **8** and **10** stand out in terms of their poor subtype selectivity profile, lacking almost any differences in their affinities towards *h*M_2_R, *h*M_4_R, *h*M_5_R and *h*M_3_R, *h*M_4_R, *h*M_5_R, respectively. Breaking the present compound series’ general trend of, at best, moderate *h*M_1_ selectivity over *h*M_5_, spiro compound **5** displays a decent 9-fold selectivity over this subtype.

Overall, we have demonstrated with the design of this *h*M_1_ preferring carbamate series that subtle structural changes can have profound effects on binding affinities and good subtype selectivity is not an unrealistic objective even in the case of orthosteric mAChR ligands.

**Figure 5:**
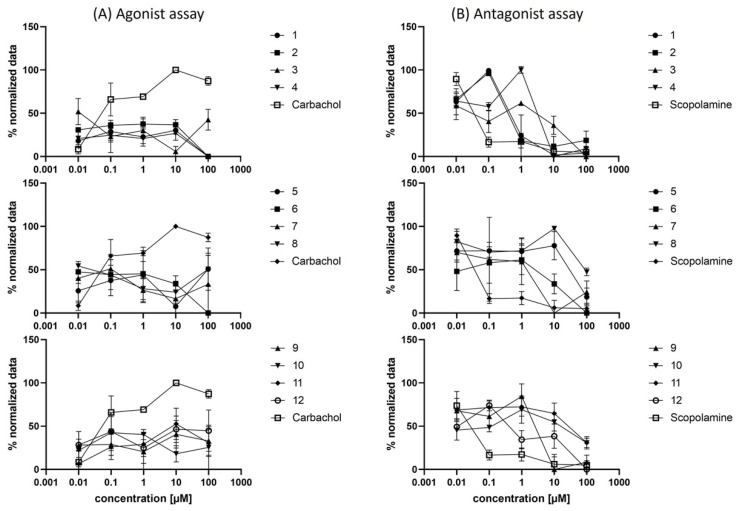
Dose-dependent Ca^2+^ mobilization induced by carbamates **1**–**12** in CHO-*h*M_1_ cells. (**A**) Agonist dose-response experiment; (**B**) Antagonist dose-response experiment with the reference agonist carbachol added at a final concentration of 20 µM.

To further assess the synthesized compounds’ functionality, i.e., to identify whether they behave in an agonistic or antagonistic fashion, CHO-*h*M_1_ cells were treated with **1**–**12** and subsequently assayed for calcium mobilization using Fluo-4 [43]. In comparison to the known mAChR agonist carbachol, none of the tested compounds showed a similar progression of the effect-concentration curve (Figure 5A); however, using scopolamine as positive control and treating the cells with **1**–**12** led to an inhibition of carbachol-induced calcium flux (Figure 5B), clearly illustrating the antagonistic binding of all tested compounds. While the raison d’être for agonistic GPCR imaging probes is critically discussed [44], antagonism can be viewed as an advantage in the realm of PET imaging as it renders the possibility of unwanted pharmacological (e.g., ligand-induced conformational change and activation of the target GPCR) and side effects unlikely.

With the promising physico–chemical property profile, the binding affinities, and the subtype selectivity profiles of the herein presented carbamate-bridged compounds **2**, **5**, and **7** in mind, the potential application as PET imaging probes should be followed up on. Furthermore, the common tertiary *N*-methyl amine moiety, which has been a structural prerequisite in our computational workflow, is assumed to allow for straightforward carbon-11 radiolabeling by utilizing the corresponding *N*-desmethyl precursors and reacting them with [^11^C]MeI [16].

## 3. Materials and Methods

### 3.1. Ligand Design

A library of 52,857 amines, created by merging in-stock primary and secondary amines from Enamine and in-stock diamines from Chemspace, was salt-stripped and filtered for cyclic aliphatic primary and secondary at least mono *N*-methyl diamines using the FILTER program from OpenEye [45]. After dropping duplicates, undefined stereocenters were enumerated using the Flipper program from OpenEye [45]. This focused selection of 331 diamine fragments was linked with a 4,4’-difluorobenzhydryl motif via a carbamate bridge, and the molecules were set to their energetically most favorable ionization state at pH 7.4 using the FixpKa program from OpenEye [46]. All resulting potential ligands were docked in the M_1_ muscarinic acetylcholine receptor crystal structure 5CXV using AutoDock Vina 1.1 with default settings [47]. The performance of the docking algorithm was validated in a re-docking experiment, in which the co-crystallized ligand’s binding pose was reproduced with an acceptable RMSD of 0.252 Å [48]. Poses whose protonated *N*-methyl moiety did not come within 5.5 Å of Asp105^3.32′^s carboxyl oxygens were removed by utilizing LigGrep as post-docking filter [49]. Docking results and the corresponding receptor-ligand interactions were analyzed with the software LigandScout 4.4.5 [22]. To visualize the spatial arrangement of such interactions, 2D and pharmacophores were generated using the same software. Docking poses were additionally visualized using PyMOL [50]. The highest ranked pose of each docked compound exhibiting an ionic interaction with Asp105^3.32^ was selected as a representative resulting in a final dataset of 129 potential ligands. Manual selection from this dataset resulted in 12 readily accessible compounds for further experimental evaluation. 

### 3.2. Chemistry

#### 3.2.1. General Considerations

Unless otherwise stated, all reagents were purchased from commercial suppliers and used as received without further purification. All reactions were conducted under an inert atmosphere of argon, and commercially available anhydrous solvents were used. Flash column chromatography was either performed on a Biotage^®^ Isolera™ One or Biotage^®^ Selekt Flash Chromatography System equipped with Biotage^®^ SNAP Ultra HP-Sphere 25 μm or Biotage^®^ Sfär HC cartridges using either HPLC grade or reagent grade solvents. Reactions were monitored by TLC on pre-coated aluminum sheets (Polygram SIL G/UV254, 0.2 mm, with fluorescent indicator; Macherey-Nagel, Düren, Germany); the spots were visualized under UV light (λ = 254 nm) and/or KMnO_4_ stain. ^1^H, ^13^C, and ^19^F NMR spectra were recorded in deuterated chloroform (CDCl_3_) at 298 K on a Bruker Avance III 400 spectrometer and are reported as follows: chemical shift δ in ppm (multiplicity, coupling constant *J* in Hz, number of protons, assignment) for ^1^H NMR spectra and chemical shift δ in ppm (assignment) for ^13^C and ^19^F spectra. For ^1^H and ^13^C NMR spectra residual solvent peaks of CDCl_3_ (δ_H_ = 7.26 ppm, δ_C_ = 77.00 ppm) were used as internal reference. ^19^F NMR spectra were referenced according to Ξ-values. The chemical shifts of all signals are reported as the center of the resonance range (Supplementary Materials, Figures S17–S40). Unless stated otherwise, full and unambiguous assignment of all resonances was performed by a combination of standard NMR techniques, such as APT, HSQC, HMBC, COSY, and NOESY experiments. IR spectra were recorded on a Bruker Alpha II FTIR spectrometer. Samples were prepared as a film by evaporation of a solution in CH_2_Cl_2_ and selected absorption bands are reported in wavenumbers (cm^−1^). HRMS spectra were recorded on a Bruker maXis 4G instrument (ESI-TOF). Melting points were measured with an Electrothermal IA9200 melting point apparatus in open glass capillaries and are uncorrected. All tested compounds exhibited ≥95% purity under the HPLC conditions reported hereafter. HPLC analyses were performed either on a Shimadzu HPLC system consisting of a degassing unit (DGU-20A3R), a liquid chromatograph (LC-20ADXR), an autosampler (SIL-20ACHT), a diode array detector (SPD-M20A), a column oven (CTO-20AC) and a communication bus module (CBM-20A) or an Agilent 1260 Infinity HPLC system consisting of an autosampler (series 1100), pump (series 1200), diode array detector (series 1100) and a radiodetector (Ramona, Elisa-Raytest). The stationary phase was an Eclipse Plus column (4.6 × 100 mm, 3.5 µm, Agilent, Santa Clara, CA, USA) and the mobile phase consisted of the following components: solvent A: 0.1% TFA in double distilled water; solvent B: 0.1% TFA in acetonitrile. Purity was measured with a gradient run starting with 10% up to 100% solvent B within 9.4 min with a flow of 1.5 mL/min as well as with an isocratic run (Supplementary Materials, Figures S5–S16).

#### 3.2.2. General Procedure for the Alkoxycarbonylation of Diamines

The following procedure was adapted from the literature [51]. In case a diamine was present in its salt form it needed to be converted to its free base by suspending it in sat. aq. Na_2_CO_3_, extracting with CH_2_Cl_2_ (3×), drying (Na_2_SO_4_), and concentrating under reduced pressure. To a stirred solution of diamine (free base, 1.0 equiv) in anhydrous DMF (0.2 M) was added CDI (1.0 equiv) in one portion at ambient temperature. The resulting reaction mixture was stirred at this temperature for 12 h. In a second ice-cooled flask, NaH (60% dispersion in mineral oil, 2.0 equiv) was added to a stirred solution of bis(4-fluorophenyl)methanol (2.0 equiv) in anhydrous DMF (0.5 M). It was stirred for 30 min at ambient temperature, then the alkoxide solution was added to the carbamoylimidazole at ambient temperature. The resulting mixture was stirred for 24 h. Then, volatiles were removed under reduced pressure and the residue was dissolved in CH_2_Cl_2_ and washed with water (2×). The organic layer was dried (Na_2_SO_4_) and concentrated under reduced pressure. The crude residue was purified via flash column chromatography to give the desired product.

Bis(4-fluorophenyl)methyl 4-methylpiperazine-1-carboxylate (**1**). Following the general procedure on a 0.25 mmol scale, 1-methylpiperazine was alkoxycarbonylated. Purification by flash column chromatography (0–10% MeOH in CH_2_Cl_2_) afforded the title compound **1** (29 mg, 34%) as a colorless solid. mp 106–108 °C. ^1^H NMR (400 MHz, CDCl_3_) δ 7.27 (m, 4H, Ph H-2,6), 7.02 (m, 4H, Ph H-3,5), 6.77 (s, 1H, CHPh_2_), 3.61 (br s, 2H, H-2,6), 3.52 (br s, 2H, H-2,6), 2.38 (m, 4H, H-3,5), 2.31 (s, 3H, NCH_3_). ^13^C NMR (100 MHz, CDCl_3_) δ 162.3 (d, *J* = 246.7 Hz, Ph C-4), 154.2 (C=O), 136.4 (d, *J* = 3.3 Hz, Ph C-1), 128.7 (d, *J* = 8.2 Hz, Ph C-2,6), 115.4 (d, *J* = 21.6 Hz, Ph C-3,5), 76.6 (CHPh_2_), 54.7 (C-3,5), 46.1 (NCH_3_), 43.8 (C-2,6). ^19^F NMR (377 MHz, CDCl_3_) δ −114.2 (m, Ph-F). IR (film) $\nu_{\max}$ 1701, 1605, 1508, 1458, 1430, 1293, 1259, 1226, 1148, 1100, 1070, 1013, 1003, 836, 572, 540. HRMS (ESI) (*m*/*z*) calcd. for C_19_H_21_F_2_N_2_O_2_ [M + H]^+^: 347.1566; found 347.1570.

Bis(4-fluorophenyl)methyl 4-methyl-1,4-diazepane-1-carboxylate (**2**). Following the general procedure on a 0.25 mmol scale, 1-methyl-1,4-diazepane was alkoxycarbonylated. Purification by flash column chromatography (0–10% MeOH in CH_2_Cl_2_) afforded the title compound **2** (34 mg, 38%) as an off-white solid. mp 64–66 °C. ^1^H NMR (400 MHz, CDCl_3_) (mixture of rotamers) δ 7.28 (m, 4H, Ph H-2,6), 7.01 (m, 4H, Ph H-3,5), 6.78 (s, 1H, CHPh_2_), 3.64 (m, 2H, H-2,7), 3.59 (m, 1H, H-2), 3.54 (m, 1H, H-7), 2.62 (m, 2H, H-3), 2.55 (m, 2H, H-5), 2.365/2.360 (s, 3H, NCH_3_). ^13^C NMR (100 MHz, CDCl_3_) (mixture of rotamers) δ 162.3 (d, *J* = 246.6 Hz, Ph C-4), 155.14/154.98 (C=O), 136.60/136.56 (m, Ph C-1), 128.70/128.65 (d, *J* = 8.2 Hz, Ph C-2,6), 115.4 (d, *J* = 21.5 Hz, Ph C-3,5), 76.5 (CHPh_2_), 58.51/58.43 (C-3), 57.38/57.12 (C-5), 46.68/46.57 (NCH_3_), 46.22/46.13 (C-2), 45.90/45.84 (C-7), 27.52/27.48 (C-6). ^19^F NMR (377 MHz, CDCl_3_) δ −114.4 (m, Ph-F). IR (film) $\nu_{\max}$ 1697, 1605, 1508, 1462, 1412, 1291, 1222, 1186, 1157, 1113, 1047, 1004, 834, 572, 543. HRMS (ESI) (*m*/*z*) calcd. for C_20_H_23_F_2_N_2_O_2_ [M + H]^+^: 361.1722; found 361.1722.

Bis(4-fluorophenyl)methyl5-methyl-2,5-diazabicyclo[2.2.1]heptane-2-carboxylate (**3**). Following the general procedure on a 0.25 mmol scale, 2-methyl-2,5-diazabicyclo[2.2.1]heptane dihydrobromide was alkoxycarbonylated. Purification by flash column chromatography (0–10% MeOH in CH_2_Cl_2_) afforded the title compound **3** (9 mg, 10%) as a pale-yellow solid. mp 81–83 °C. ^1^H NMR (400 MHz, CDCl_3_) (mixture of rotamers) δ (major) 7.28 (m, 4H, Ph H-2,6), 7.01 (m, 4H, Ph H-3,5), 6.75 (s, 1H, CHPh_2_), 4.40 (m, 1H, H-1), 3.72 (m, 1H, H-3), 3.49 (m, 1H, H-4), 3.35 (m, 1H, H-3), 2.85 (m, 1H, H-6), 2.76 (m, 1H, H-6), 2.44 (s, 3H, NCH_3_), 1.92 (m, 1H, H-7), 1.74 (m, 1H, H-7); (minor) 7.28 (m, 4H, Ph H-2,6), 7.01 (m, 4H, Ph H-3,5), 6.79 (s, 1H, CHPh_2_), 4.46 (m, 1H, H-1), 3.62 (m, 1H, H-3), 3.48 (m, 1H, H-4), 3.23 (m, 1H, H-3), 3.02 (m, 1H, H-6), 2.55 (m, 1H, H-6), 2.42 (s, 3H, NCH_3_), 1.95 (m, 1H, H-7), 1.77 (m, 1H, H-7). ^13^C NMR (100 MHz, CDCl_3_) (mixture of rotamers) δ (major) 162.3 (d, *J* = 246.7, Ph C-4), 153.5 (C=O), 136.50/136.47 (Ph C-1), 128.9–128.5 (m, Ph C-2,6), 115.4 (d, *J* = 21.6 Hz, Ph C-3,5), 76.1 (CHPh_2_), 63.1 (C-4), 61.39 (C-6), 57.8 (C-1), 49.6 (C-3), 41.2 (NCH_3_), 34.9 (C-7); (minor) 162.3 (d, *J* = 246.7, Ph C-4), 153.4 (C=O), 136.50/136.47 (Ph C-1), 128.9–128.5 (m, Ph C-2,6), 115.4 (d, *J* = 21.6 Hz, Ph C-3,5), 76.2 (CHPh_2_), 62.3 (C-4), 61.45 (C-6), 58.1 (C-1), 48.9 (C-3), 40.4 (NCH_3)_, 36.2 (C-7). ^19^F NMR (377 MHz, CDCl_3_) δ −114.4–−114.2 (m, Ph-F). IR (film) $\nu_{\max}$ 1700, 1605, 1508, 1404, 1331, 1222, 1181, 1157, 1131, 1087, 837, 571, 545. HRMS (ESI) (*m*/*z*) calcd. for C_20_H_21_F_2_N_2_O_2_ [M + H]^+^: 359.1566; found 359.1583.

Bis(4-fluorophenyl)methyl (1*R*,5*S*)-8-methyl-3,8-diazabicyclo[3.2.1]octane-3 carboxylate (**4**). Following the general procedure on a 0.25 mmol scale, (1*R*,5*S*)-8-methyl-3,8-diazabicyclo[3.2.1]octane dihydrochloride was alkoxycarbonylated. Purification by flash column chromatography (0–10% MeOH in CH_2_Cl_2_) afforded the title compound **4** (23 mg, 25%) as a colorless solid. mp 112–115 °C. ^1^H NMR (400 MHz, CDCl_3_) δ 7.26 (m, 4H, Ph H-2,6), 7.02 (m, 4H, Ph H-3,5), 6.76 (s, 1H, CHPh_2_), 3.78 (m, 4H, H-2,4), 3.25 (m, 2H, H-6,7), 3.12 (m, 2H, H-1,5), 3.10 (m, 2H, H-6,7), 2.29 (s, 3H, NCH_3_). ^13^C NMR (100 MHz, CDCl_3_) (mixture of rotamers) δ 162.3 (d, *J* = 246.7 Hz, Ph C-4), 155.4 (C=O), 136.6/136.3 (d, *J* = 3.1 Hz, Ph C-1), 128.2 (d, *J* = 8.2 Hz, Ph C-2,6), 115.45/115.41 (d, *J* = 21.6 Hz, Ph C-3,5), 76.6 (CHPh_2_), 60.4/60.2 (C-1,5), 50.2/50.0 (C-2,4), 40.7 (NCH_3_), 24.9/24.6 (C-6,7).^19^F NMR (377 MHz, CDCl_3_) δ −114.31 (m, Ph-F), −114.25 (m, Ph-F). IR (film) $\nu_{\max}$ 2943, 1699, 1605, 1508, 1428, 1243, 1222, 1156, 1132, 1091, 1069, 983, 837, 571, 540. HRMS (ESI) (*m*/*z*) calcd. for C_21_H_23_F_2_N_2_O_2_ [M + H]^+^: 373.1722; found 373.1720.

Bis(4-fluorophenyl)methyl 2-methyl-2,6-diazaspiro[3.4]octane-6-carboxylate (**5**). Following the general procedure on a 0.25 mmol scale, 2-methyl-2,6-diazaspiro[3.4]octane was alkoxycarbonylated. Purification by flash column chromatography (0–10% MeOH in CH_2_Cl_2_) afforded the title compound **5** (24 mg, 26%) as a pale-yellow oil. ^1^H NMR (400 MHz, CDCl_3_) (mixture of rotamers) δ (major) 7.29 (m, 4H, Ph C-2,6), 7.01 (m, 4H, Ph C-3,5), 6.75 (br s, 1H, CHPh_2_), 3.63 (s, 2H, C-5), 3.39 (t, *J* = 7.0 Hz, 2H, H-7), 3.23 (m, 2H, H-1,3), 3.17 (m, 2H, H-1,3), 2.353 (s, 3H, NCH_3_), 2,01 (t, *J* = 7.0 Hz, 2H, H-8); (minor) 7.29 (m, 4H, Ph C-2,6), 7.01 (m, 4H, Ph C-3,5), 6.75 (br s, 1H, CHPh_2_), 3.51 (t, *J* = 7.0 Hz, 2H, H-7), 3.46 (s, 2H, H-5), 3.29 (m, 2H, H-1,3), 3.14 (m, 2H, H-1,3), 2.346 (s, 3H, NCH_3_), 2.13 (t, *J* = 7.0 Hz, 2H, H-8). ^13^C NMR (100 MHz, CDCl_3_) (mixture of rotamers) δ (major) 162.2 (d, *J* = 246.6 Hz, Ph C-4), 153.73 (C=O), 136.6 (m, Ph C-1), 128.7 (d, *J* = 8.1 Hz, Ph C-2,6), 115.4 (d, *J* = 21.6 Hz, Ph C-3,5), 76.13 (CHPh_2_), 65.48 (C-1,3), 55.39 (C-5), 45.79 (NCH_3_), 44.90 (C-7), 40.80 (C-4), 35.87 (C-8); (minor) 162.2 (d, *J* = 246.6 Hz, Ph C-4), 153.69 (C=O), 136.6 (m, Ph C-1), 128.7 (d, *J* = 8.1 Hz, Ph C-2,6), 115.4 (d, *J* = 21.6 Hz, Ph C-3,5), 76.09 (CHPh_2_), 65.32 (C-1,3), 55.32 (C-5), 45.77 (NCH_3_), 44.63 (C-7), 39.77 (C-4), 34.96 (C-8). ^19^F NMR (377 MHz, CDCl_3_) δ. −114.4 (m, Ph-F). IR (film) $\nu_{\max}$ 1699, 1605, 1508, 1403, 1222, 1185, 1157, 1128, 1095, 1083, 833, 764, 573, 546. HRMS (ESI) (*m*/*z*) calcd. for C_21_H_23_F_2_N_2_O_2_ [M + H]^+^: 373.1722; found 373.1742.

Bis(4-fluorophenyl)methyl (1-methylpiperidin-4-yl)carbamate (**6**). Following the general procedure on a 0.25 mmol scale, 1-methylpiperidin-4-amine was alkoxycarbonylated. Purification by flash column chromatography (0–10% MeOH in CH_2_Cl_2_) afforded the title compound **6** (21 mg, 22%) as a colorless solid. mp 159–161 °C. ^1^H NMR (400 MHz, CDCl_3_) δ 7.27 (m, 4H, Ph H-2,6), 7.02 (m, 4H, Ph H-3,5), 6.73 (s, 1H, CHPh_2_), 4.78 (d, *J* = 7.8 Hz, 1H, NH), 3.50 (m, 1H, H-4), 2.77 (m, 2H, H-2,6), 2.27 (s, 3H, NCH_3_), 2.07 (m, 2H, H-2,6), 1.93 (m, 2H, H-3,5), 1.49 (m, 2H, H-3,5). ^13^C NMR (100 MHz, CDCl_3_) δ 162.3 (d, *J* = 246.8 Hz, Ph C-4), 154.6 (C=O), 136.3 (d, *J* = 3.1 Hz, Ph C-1), 128.8 (d, *J* = 8.2 Hz, Ph C-2,6), 115.4 (d, *J* = 21.6 Hz, Ph C-3,5), 76.0 (CHPh_2_), 54.3 (C-2,6), 47.9 (C-4), 46.1 (NCH_3_), 32.4 (C-3,5). ^19^F NMR (377 MHz, CDCl_3_) δ −114.2 (m, Ph-F). IR (film) $\nu_{\max}$ 1702, 1508, 1272, 1222, 1185, 1156, 1096, 1037, 1008, 832, 771, 566, 540. HRMS (ESI) (*m*/*z*) calcd. for C_20_H_23_F_2_N_2_O_2_ [M + H]^+^: 361.1722; found 361.1725.

Bis(4-fluorophenyl)methyl (1-methylpiperidin-3-yl)carbamate (**7**). Following the general procedure on a 0.25 mmol scale, 1-methylpiperidin-3-amine was alkoxycarbonylated. Purification by flash column chromatography (0–10% MeOH in CH_2_Cl_2_) afforded the title compound **7** (31 mg, 34%) as a colorless solid. mp 118–119 °C. ^1^H NMR (400 MHz, CDCl_3_) δ 7.28 (m, 4H, Ph H-2,6), 7.01 (m, 4H, Ph H-3,5), 6.73 (s, 1H, CHPh_2_), 5.50 (br s, 1H, NH), 3.81 (m, 1H, H-3), 2.48 (m, 1H, H-6), 2.41 (m, 2H, H-2), 2.24 (s, 3H, NCH_3_), 2.21 (m, 1H, H-6), 1.72 (m, 1H, H-5), 1.56 (m, 1H, H-5), 1.55 (m, 2H, H-4). ^13^C NMR (100 MHz, CDCl_3_) δ 162.3 (d, *J* = 246.6 Hz, Ph C-4), 154.6 (C=O), 136.5 (d, *J* = 3.1 Hz, Ph C-1), 128.7 (t, *J* = 7.7 Hz, Ph C-2,6), 115.4 (d, *J* = 21.5 Hz, Ph C-3,5), 75.8 (CHPh_2_), 60.3 (C-2), 55.7 (C-6), 46.7 (C-3), 46.3 (NCH_3_), 28.6 (C-4), 21.8 (C-5). ^19^F NMR (377 MHz, CDCl_3_) δ −114.4 (m, Ph-F). IR (film) $\nu_{\max}$ 2939, 1710, 1605, 1508, 1223, 1186, 1157, 1098, 1066, 1037, 1014, 833, 540. HRMS (ESI) (*m*/*z*) calcd. for C_20_H_23_F_2_N_2_O_2_ [M + H]^+^: 361.1722; found 361.1722.

Bis(4-fluorophenyl)methyl ((1*R*,3*s*,5*S*)-9-methyl-9-azabicyclo[3.3.1]nonan-3-yl)carbamate (**8**). Following the general procedure on a 0.25 mmol scale, (1*R*,5*R*)-9-methyl-9-azabicyclo[3.3.1]nonan-3-amine was alkoxycarbonylated. Purification by flash column chromatography (0–10% MeOH in CH_2_Cl_2_) afforded the title compound **8** (25 mg, 25%) as a colorless solid. mp 144–147 °C. ^1^H NMR (400 MHz, CDCl_3_) δ 7.27 (m, 4H, Ph H-2,6), 7.01 (m, 4H, Ph H-3,5), 6.75 (br s, 1H, CHPh_2_), 4.69 (br d, *J* = 7.0 Hz, 1H, NH), 4.07 (m, 1H, H-3), 3.05 (m, 2H, H-1,5), 2.47 (s, 3H, NCH_3_), 2.40 (m, 2H, H-2,4), 1.93 (m, 2H, H-6,8), 1.91 (m, 1H, H-7), 1.49 (m, 1H, H-7), 1.01 (m, 2H, H-6,8). ^13^C NMR (100 MHz, CDCl_3_) δ 162.3 (d, *J* = 246.6 Hz, Ph C-4), 154.5 (C=O), 136.4 (br s, Ph C-1), 128.8 (d, *J* = 8.2 Hz, Ph C-2,6), 115.4 (d, *J* = 21.5 Hz, Ph C-3,5), 75.6 (CHPh_2_), 51.3 (C-1,5), 42.9 (C-3), 40.2 (NCH_3_), 33.0 (C-2,4), 24.3 (C-6,8), 14.1 (C-7). ^19^F NMR (377 MHz, CDCl_3_) δ −114.4 (m, Ph-F). IR (film) $\nu_{\max}$ 2926, 1702, 1509, 1288, 1262, 1224, 1156, 1046, 1014, 833. HRMS (ESI) (*m*/*z*) calcd. for C_23_H_27_F_2_N_2_O_2_ [M + H]^+^: 401.2035; found 401.2045.

Bis(4-fluorophenyl)methyl (1-methylpyrrolidin-3-yl)carbamate (**9**). Following the general procedure on a 0.25 mmol scale, 1-methylpyrrolidin-3-amine was alkoxycarbonylated. Purification by flash column chromatography (0–20% MeOH in CH_2_Cl_2_) afforded the title compound **9** (16 mg, 19%) as an off-white solid. mp 84–86 °C. ^1^H NMR (400 MHz, CDCl_3_) δ 7.26 (m, 4H, Ph H-2,6), 7.00 (m, 4H, Ph H-3,5), 6.71 (s, 1H, CHPh_2_), 5.51 (d, *J* = 8.0 Hz, 1H, NH), 2.93 (m, 1H, H-5), 2.69 (m, 1H, H-2), 2.53 (m, 1H, H-2), 2.37 (s, 3H, NCH_3_), 2.29 (m, 1H, H-4), 2.27 (m, 1H, H-5), 1.67 (m, 1H, H-4). ^13^C NMR (100 MHz, CDCl_3_) (mixture of rotamers) δ 162.3 (d, *J* = 246.7 Hz, Ph C-4), 154.8 (C=O), 136.3 (m, Ph C-1), 128.73/128.69 (d, *J* = 8.2 Hz, Ph C-2,6), 115.4 (d, *J* = 21.6 Hz, Ph C-3,5), 76.0 (CHPh_2_), 62.9 (C-2), 54.8 (C-5), 50.9 (C-3), 41.6 (NCH3), 33.0 (C-4). ^19^F NMR (377 MHz, CDCl_3_) δ −114.3 (m, Ph-F). IR (film) $\nu_{\max}$ 1713, 1605, 1508, 1292, 1253, 1224, 1186, 1157, 1085, 1061, 1014, 998, 833. HRMS (ESI) (*m*/*z*) calcd. for C_19_H_21_F_2_N_2_O_2_ [M + H]^+^: 347.1566; found 347.1578.

Bis(4-fluorophenyl)methyl ((1-methylpyrrolidin-3-yl)methyl)carbamate (**10**). Following the general procedure on a 0.25 mmol scale, (1-methylpyrrolidin-3-yl)methanamine dihydrochloride was alkoxycarbonylated. Purification by flash column chromatography (0–10% MeOH in CH_2_Cl_2_) afforded the title compound **10** (26 mg, 29%) as a yellow semi-solid. ^1^H NMR (400 MHz, CDCl_3_) δ 7.28 (m, 4H, Ph H-2,6), 7.02 (m, 4H, Ph H-3,5), 6.73 (s, 1H, CHPh_2_), 5.48 (br s, 1H, NH), 3.24 (br s, 2H, NHCH_2_), 2.90 (m, 1H, H-5), 2.75 (m, 1H, H-2), 2.70 (m, 1H, H-5), 2.66 (m, 1H, H-2), 2.56 (m, 1H, H-3), 2.50 (s, 3H, NCH_3_), 2.08 (m, 1H, H-4), 1.63 (m, 1H, H-4). ^13^C NMR (100 MHz, CDCl_3_) δ 162.3 (d, *J* = 246.8 Hz, Ph C-4), 155.7 (C=O), 136.3 (d, *J* = 3.0 Hz, Ph C-1), 128.8 (dd, *J* = 8.2, 6.3 Hz, Ph C-2,6), 115.4 (d, *J* = 21.6 Hz, Ph C-3,5), 76.0 (CHPh_2_), 60.1 (C-2), 55.9 (C-5), 45.6 (NHCH_2_), 42.0 (NCH_3_), 37.6 (C-3), 28.6 (C-4). ^19^F NMR (377 MHz, CDCl_3_) δ −114.2 (m, Ph-F). IR (film) $\nu_{\max}$ 1704, 1605, 1507, 1222, 1185, 1156, 1134, 1100, 832, 573, 541. HRMS (ESI) (*m*/*z*) calcd. for C_20_H_23_F_2_N_2_O_2_ [M + H]^+^: 361.1722; found 361.1756.

Bis(4-fluorophenyl)methyl (2-(1-methylpyrrolidin-2-yl)ethyl)carbamate (**11**). Following the general procedure on a 0.25 mmol scale, 2-(1-methylpyrrolidin-2-yl)ethan-1-amine was alkoxycarbonylated. Purification by flash column chromatography (0–10% MeOH in CH_2_Cl_2_) afforded the title compound **11** (26 mg, 28%) as a pale-yellow oil. ^1^H NMR (400 MHz, CDCl_3_) δ 7.26 (m, 4H, Ph H-2,6), 7.00 (m, 4H, Ph H-3,5), 6.74 (s, 1H, CHPh_2_), 5.72 (m, 1H, NH), 3.25 (m, 2H, NHCH_2_), 3.05 (m, 1H, H-5), 2.28 (m, 3H, NCH_3_), 2.16 (m, 1H, H-2), 2.12 (m, 1H, H-5), 1.87 (m, 1H, H-3), 1.75 (m, 1H, NHCH_2_CH_2_), 1.69 (m, 2H, H-4), 1.56 (m, 1H, NHCH_2_CH_2_), 1.52 (m, 1H, H-3). ^13^C NMR (100 MHz, CDCl_3_) (mixture of rotamers) δ 162.22/162.20 (d, *J* = 246.6 Hz, Ph C-4), 155.4 (C=O), 136.4 (d, *J* = 3.1 Hz, Ph C-1), 128.74/128.67 (d, *J* = 8.2 Hz, Ph C-2,6), 115.3 (d, *J* = 21.6 Hz, Ph C-3,5), 75.7 (br, CHPh_2_), 64.3 (C-2), 57.0 (C-5), 40.4 (NCH_3_), 38.4 (NHCH_2_), 32.1 (NHCH_2_CH_2_), 29.6 (C-3), 22.1 (C-4). ^19^F NMR (377 MHz, CDCl_3_) δ −114.3 (m, Ph-F). IR (film) $\nu_{\max}$ 2946, 1714, 1605, 1508, 1224, 1185, 1157, 1130, 1015, 1000, 834, 572, 541. HRMS (ESI) (*m*/*z*) calcd. for C_21_H_25_F_2_N_2_O_2_ [M + H]^+^: 375.1879; found 375.1915. 

Bis(4-fluorophenyl)methyl ((4-methylmorpholin-3-yl)methyl)carbamate (**12**). Following the general procedure on a 0.25 mmol scale, (4-methylmorpholin-3-yl)methanamine was alkoxycarbonylated. Purification by flash column chromatography (0–10% MeOH in CH_2_Cl_2_) afforded the title compound **12** (27 mg, 29%) as a pale-yellow solid. mp 77–78 °C. ^1^H NMR (400 MHz, CDCl_3_) δ 7.28 (m, 4H, Ph H-2,6), 7.01 (m, 4H, Ph H-3,5), 6.73 (s, 1H, CHPh_2_), 5.37 (br s, 1H, NH), 3.76 (m, 1H, H-6), 3.71 (m, 1H, H-2), 3.57 (m, 1H, H-6), 3.36 (m, 1H, H-2), 3.28 (br s, 2H, NHCH_2_), 2.67 (m, 1H, H-5), 2.36 (m, 1H, H-5), 2.28 (s, 3H, NCH_3_), 2.25 (m, 1H, H-3). ^13^C NMR (100 MHz, CDCl_3_) (mixture of rotamers) δ 162.2 (d, *J* = 246.8 Hz), 155.5 (C=O), 136.2 (m, Ph C-1), 128.71/128.64 (d, *J* = 8.0 Hz, Ph C-2,6), 115.35/115.34 (d, *J* = 21.6 Hz, Ph C-3,5), 68.7 (C-2), 66.7 (C-6), 60.4 (C-3), 54.8 (C-5), 42.4 (NCH_3_), 38.9 (NHCH_2_). ^19^F NMR (377 MHz, CDCl_3_) δ −114.1 (m, Ph-F). IR (film) $\nu_{\max}$ 2961, 1722, 1606, 1509, 1225, 1186, 1157, 1124, 1099, 1075, 1046, 1015, 992, 835, 541. HRMS (ESI) (*m*/*z*) calcd. for C_20_H_23_F_2_N_2_O_3_ [M + H]^+^: 377.1671; found 377.1701.

### 3.3. High Throughput HPLC-logD

The high throughput HPLC-logD values were determined as published previously using the Shimadzu HPLC system described above equipped with an apHERA C18 column (10 × 6 mm, 5 µm, Supelco, Bellefonte, PA, USA) [27,30]. Briefly, a mixture of toluene (≥98%, Sigma-Aldrich, St. Louis, MO, USA) and triphenylene (≥99.9%, Carl Roth, Karlsruhe, Germany) was used as internal standard. Each sample was dissolved in the internal standard mixture. Using gradient elution, the injection volume was set to 7 µL, the flow rate was 1.5 mL/min, and the mobile phase consisted of a mixture of methanol and 0.01 M sodium phosphate buffer pH 7.4. The HPLC-logD values were derived from the measured retention times following the previously published equation [27,30].

### 3.4. Biological Evaluation

#### 3.4.1. Materials and Methods

Reagents and cell culture media were purchased from Sigma-Aldrich (St. Louis, MO, USA) and Life Technologies (Waltham, MA, USA) unless specified otherwise. Commercially obtained compounds had >98% purity. [*N*-methyl-^3^H]scopolamine methyl chloride ([^3^H]NMS) (specific activity 85.4 Ci/mmol) was purchased from PerkinElmer (Waltham, MA, USA). All analytical buffers were prepared in double distilled water (GFL water still 2004). Protease Inhibitor Cocktail powder (P2714-1BTL, Sigma-Aldrich, St. Louis, MO, USA) was dissolved in 10 mL water and used as such. Stock solutions of all compounds were prepared in pure DMSO.

#### 3.4.2. Cell Culture

Chinese hamster ovary (CHO-K1) cells stably expressing the hM_1_-hM_5_ receptors were obtained from Missouri University of Science and Technology cDNA Resource Center (Cell Catalog#: CEM1000000, CEM2000000, CEM3000000, CEM4000000, CEM5000000) and cultivated in Gibco™ Ham’s F-12 Nutrient Mixture supplemented with 10% (*v*/*v*) Gibco™ FBS, 250 mg/mL Geneticin^®^ (G418, Thermo Fisher, Waltham, MA, USA), and L-glutamine (1%; 200 nM) at 37 °C in a 5% CO_2_ humidified atmosphere. Gibco™ Trypsin-EDTA (0.05%) was used for passaging cells.

#### 3.4.3. Cell Viability (MTT Assay)

Cytotoxicity was determined by means of a colorimetric microculture assay. For this purpose, CHO-*h*M_1_ cells were harvested from culture flasks by trypsinization and seeded into 96-well microculture plates (Corning^®^, Corning, NY, USA) in densities of 4000 cells/well (100 µL/well). After a 24 h preincubation, cells were exposed in triplicates for each concentration level to dilutions of the test compounds (**1**–**12**) in complete culture medium (100 µL/well) for 72 h. At the end of the exposure period, the compound solutions were replaced with 100 µL of non-supplemented RPMI 1640 medium and 3-(4,5-dimethylthiazol-2-yl)-2,5-diphenyltetrazoliumbromid (MTT reagent in PBS, 5 mg/mL) mixed in a 6:1 ratio. After incubation for 4 h, the medium was removed, and the formazan product was solved in DMSO (100 µL/well). Optical densities at 490 nm were measured with a microplate reader (Tecan Infinite^®^ 200 PRO, Männedorf, Switzerland) using a reference wavelength of 690 nm to correct for unspecific absorption. The quantity of viable cells was normalized to untreated controls.

#### 3.4.4. Stability in Cell Culture Media

The stabilities of **3** and **7** were measured using the HPLC gradient method as described for the purity determination. 4 µL of a test compound’s stock solution in DMSO was diluted with fully supplemented RPMI1640 cell culture medium (10% FBS, 1% L-glutamine, without antibiotics). The stability was measured at ambient temperature and the column oven was set to 20 °C. The samples were measured at 0, 30, 60, 120 min and 24 h.

#### 3.4.5. Radioligand Binding Experiments

Cell membranes bearing *h*M_1_-*h*M_5_ receptors were prepared as described previously [16]. Briefly, stably transfected CHO-K1 cells were grown to at least 80% confluency in T175 flasks, washed with ice-cold DPBS, and scraped into a mixture of ice-cold of 2 mL 10 mM Tris-HCl, 1 mM EDTA-buffer, pH 7.4 and 200 µL protease inhibitor. A cell homogenate was prepared by passing the cell suspension through a G29 needle. The cell homogenates corresponding to two T175 flasks were combined and subsequently centrifuged (10 min, 1000× *g*, 4 °C). Ultracentrifugation of the supernatant (1 h, 100,000× *g*, 4 °C) yielded a membrane pellet, which was suspended in 250 µL 50 mM Tris-HCl buffer, pH 7.4 and stored at −80 °C. 

Inhibition constants (*K*_i_) were determined by means of a competitive radioligand binding assay using 50 mM Tris-HCl, 10 mM MgCl_2_, 1 mM EDTA, pH 7.4 as assay buffer as described previously [42]. 5 µL of test compound (**1**–**12**) in DMSO, 50 µL of [^3^H]NMS in assay buffer and 445 µL of membrane suspension in assay buffer were incubated for 90 min at 23 °C in PP tubes. Maximum binding was measured by using 5 µL DMSO, and nonspecific binding was measured by using 5 µL of 1 µM scopolamine in DMSO. The effective concentration of [^3^H]NMS was 0.2 nM, 0.3 nM, 0.8 nM, 0.2 nM, and 1 nM for M_1_–M_5_ and 4–30 µg membrane was used per tube. The membrane-bound radioactivity was recovered by filtration through Whatman™ GF/B glass fibre filters pre-soaked in aqueous 0.1% PEI using an M-36 tygon tubed cell harvester (Brandel^®^, Gaithersburg, MD, USA). Membranes were washed 3 times with ice-cold washing buffer (50 mM Tris, pH 7.4) before being dried, transferred to 2 mL scintillation cocktail (UltimaGold™, high flashpoint LSC cocktail, PerkinElmer, Waltham, MA, USA) and counted in a β-counter (Hidex TDCR Liquid Scintillation Counter in CPM mode). IC_50_ values were calculated by a variable slope logistic regression using at least five distinct concentrations of test compounds, pipetted in triplicates. *K*_i_ values were then calculated with the help of the Cheng–Prusoff equation using the following *K*_D_ values of [^3^H]NMS for *h*M_1_–*h*M_5_: 0.18, 0.24, 0.23, 0.10, and 0.35 nM.

#### 3.4.6. Fluo-4 Calcium Assay for Agonist-Antagonist Discrimination

For the Fluo-4 Direct™ Assay Kit (Invitrogen, Waltham, MA, USA), 100 µL of a 5 × 105 cells/mL suspension of CHO-hM_1_ cells were seeded in black clear bottom 96-well plates (Corning^®^, Corning, NY, USA). After settling of the cells for 24 h, the kit was used according to the manufacturer’s protocol. In detail, the medium was removed, and 50 µL of Hanks’ Balanced Salt Solution (HBSS) was added, followed by 50 µL of the Fluo-4 buffer solution (including probenecid). The 96-well plates were incubated for 60 min at 37 °C in the dark. For the agonist assay, 100 µL of a double-concentrated dilution series of carbachol (positive control) and compounds **1**–**12** were added with the end concentration of 100, 10, 1, 0.1, 0.01 and 0 µM. The relative fluorescence was measured with an excitation wavelength of 494 nm and an emission wavelength of 516 nm. For the antagonist assay, 50 µL of a 4-fold concentrated dilution series of scopolamine hydrochloride (positive control) and compounds **1**–**12** were added. Subsequently, an 80 µM stock solution of carbachol was added to all wells, and the relative fluorescence was measured with an excitation wavelength of 494 nm and an emission wavelength of 516 nm. Stock solutions of the compounds were in DMSO with a final concentration not exceeding 1% of DMSO. 

#### 3.4.7. Data Analysis and Statistics

Data analysis in general was performed using Prism 9.00 (GraphPad Software, San Diego, CA, USA) or Microsoft Excel^®^ 365. Data are presented as means ± standard deviation (SD) for at least 3 independent experiments unless indicated otherwise. 

## 4. Conclusions

In summary, we have identified a series of *h*M_1_R selective orthosteric antagonists through a systematic docking campaign making use of a focused diamine library. Starting from 4-FBA as parent compound and replacing its 1,2,3,6-tetrahydropyridine moiety with primary or secondary amines attached to an aliphatic tertiary *N*-methyl amine enclosed in a cyclic structure delivered a set of carbamate-bridged compounds, displaying a promising subtype selectivity and affinity profile. In particular, the exceptional and good subtype selectivity of **2** and **5** and **7**, respectively and their attractive physico–chemical properties pointing towards brain permeation, motivated us to initiate further studies to clarify their potential in PET imaging. Additionally, studies are underway to better understand the enantiospecific affinity and selectivity profiles of the enantiomers of **7**, allowing us to proceed in radiolabeling studies with only one potentially superior isomer, which will be reported in due course.

## Figures and Tables

**Table 1:** General synthetic route towards carbamates **1**–**12**.

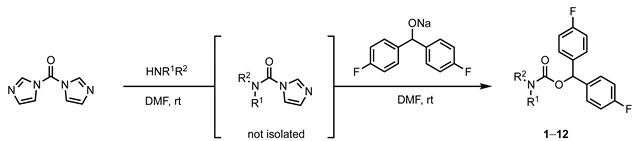					
Cmpd.	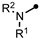	Yield ^1^ (%)	Cmpd.	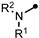	Yield ^1^ (%)
**1**	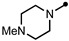	34	**7**	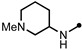	34
**2**	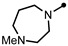	38	**8**	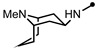	25
**3**	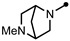	10	**9**	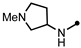	19
**4**	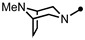	25	**10**	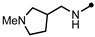	29
**5**	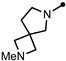	26	**11**	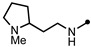	28
**6**	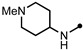	22	**12**	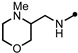	29

^1^ All yields are isolated yields.

**Table 2:** Physico–chemical properties and BBB transport parameters of carbamates **1**–**12**.

	Physico–Chemical Properties				BBB Transport Parameters	
Cmpd.	HPLC-logD	logD ^1^	tPSA ^2^ (Å^2^)	p*K*a ^3,4^	logBB ^4^	logPS ^4^
**1**	3.16 ± 0.01	3.14	32.78	6.8 ± 0.1	0.45	−1.2
**2**	3.20 ± 0.02	3.19	32.78	7.5 ± 0.1	0.37	−1.2
**3**	2.69 ± 0.01	1.37	32.78	9.5 ± 0.2	0.51	−1.6
**4**	3.28 ± 0.03	1.92	32.78	9.0 ± 0.2	0.62	−1.5
**5**	2.69 ± 0.01	2.08	32.78	9.6 ± 0.2	0.96	−1.4
**6**	3.25 ± 0.04	2.86	41.57	8.6 ± 0.1	0.50	−1.5
**7**	2.2 ± 0.2	2.44	41.57	9.4 ± 0.1	0.53	−1.5
**8**	3.21 ± 0.03	2.31	41.57	10.9 ± 0.4	0.99	−1.5
**9**	2.8 ± 0.1	1.77	41.57	9.6 ± 0.4	0.45	−1.7
**10**	2.69 ± 0.01	1.12	41.57	10.2 ± 0.4	0.39	−1.7
**11**	2.5 ± 0.3	1.39	41.57	10.3 ± 0.4	0.54	−1.6
**12**	2.82 ± 0.04	3.03	50.80	7.0 ± 0.4	0.23	−1.4

^1^ Calculated for pH 7.4 using ACD/Percepta [34]. ^2^ Calculated using LigandScout [22]. ^3^ The value corresponds to the tertiary *N*-methyl amine functionality. ^4^ Calculated using ACD/Percepta [34].

**Table 3:** Inhibition of [^3^H]NMS binding in CHO-*h*M_1-5_ cell membrane preparations and subtype selectivity profiles.

	Affinity: *K*_i_ ± SD (nM)					x-Fold Selectivity for *h*M_1_ vs. *h*M_x_^1^			
Cmpd.	*h*M_1_	*h*M_2_	*h*M_3_	*h*M_4_	*h*M_5_	*h*M_2_	*h*M_3_	*h*M_4_	*h*M_5_
**1**	15.2 ± 3.6	>1000 ^2^	225.6 ± 85.2	54.8 ± 20.5	50.6 ± 3.9	>66	14.8	3.6	3.3
**2**	1.2 ± 0.4	227.2 ± 85.9	28.4 ± 10.7	14.4 ± 5.5	4.8 ± 1.6	189.3	23.7	12.0	4.0
**3**	33.1 ± 8.1	>1000 ^2^	357.8 ± 83.0	115.1 ± 51.0	68.0 ± 22.1	>30	10.8	3.5	2.1
**4**	16.5 ± 2.8	849.5 ± 39.8	141.6 ± 24.2	19.6 ± 5.5	41.8 ± 14.8	51.5	8.6	1.2	2.5
**5**	24.9 ± 6.2	>1000 ^2^	164.5 ± 37.5	150.3 ± 52.9	230.8 ± 25.7	>40	6.6	6.0	9.3
**7**	1.22 ± 0.06	32.8 ± 11.4	16.1 ± 4.5	6.2 ± 2.1	3.7 ± 1.3	27.3	13.4	5.2	3.1
**8**	474.6 ± 88.5	623.9 ± 104.3	>1000 ^2^	562.9 ± 73.4	521.0 ± 172.7	1.3	>2	1.2	1.1
**9**	67.8 ± 5.4	721.9 ± 101.19	181.2 ± 68.1	143.8 ± 37.3	64.5 ± 22.8	10.6	2.7	2.1	1.0
**10**	238.7 ± 67.9	>1000 ^2^	276.9 ± 45.4	238.2 ± 103.6	295.2 ± 32.8	>4	1.2	1.0	1.2

^1^ The selectivity is calculated as the ratio of the *K*_i_ values, i.e., *h*M_x_/*h*M_1_. ^2^ Value derived from two independent experiments carried out in triplicate.

## Data Availability

Data is contained within the article and Supplementary Material.

## Supplementary Materials

The following are available online at https://www.mdpi.com/article/10.3390/ph15020248/s1, Figure S1: Docking poses for compounds **1**, **3**–**6**, and **8**–**12** (carbons in yellow) in the orthosteric binding site of M_1_ (PDB 5CXV) and the corresponding 2D pharmacophores. In case of chiral secondary carbamates, only one enantiomer is shown, Figure S2: Superimposed docking poses for the enantiomeric pairs of **7** and **9**–**12**, Figure S3: Stability of 3 and 7 in a fully supplemented RPMI1640 cell culture medium at 20 °C. Error bars represent the standard deviation, Figure S4: Concentration-dependent cell viability of **1**–**12** assessed in living CHO-*h*M_1_ cells using an MTT assay. Error bars represent the standard deviation, Figures S5–S16: Isocratic HPLC chromatograms of **1**–**12**. Figures S17–S40: ^1^H and ^13^C spectra of **1**–**12**, Table S1: Percent displacements of [^3^H]NMS binding on cell membranes derived from CHO-K1 cells expressing *h*M_x_ receptors at ligand concentrations corresponding to a *K*_i_ value of 1 µM according to the Cheng–Prusoff Equation.
