# Supplementary material for: Design, Synthesis, and Biological Evaluation of 4,4’-Difluorobenzhydrol Carbamates as Selective M1 Antagonists
Source: Pharmaceuticals (Basel). 2022 Feb 18;15(2):248. doi: 10.3390/ph15020248 (PMC8879200; doi:10.3390/ph15020248)
Supplement: Supplementary file 1 [file pharmaceuticals-15-00248-s001.zip › pharmaceuticals-1585872-supplementary.pdf]

pharmaceuticals

Supporting Information

Design, Synthesis, and Biological Evaluation of 4,4'-Difluorobenzhydrol Carbamates as Selective M1 Antagonists

Jonas Kilian [1], Marius Ozenil [1], Marlon Millard [2], Dorka Fürtös [2], Verena Maisetschläger [2], Wolfgang Holzer [2], Wolfgang Wadsak [1,3], Marcus Hacker [1], Thierry Langer [2] and Verena Pichler [2,*]

1 Department of Biomedical Imaging and Image-guided Therapy, Division of Nuclear Medicine, Medical University of Vienna, 1090 Vienna, Austria; jonas.kilian@meduniwien.ac.at (J.K.); marius.ozenil@meduniwien.ac.at (M.O.); wolfgang.wadsak@meduniwien.ac.at (W.W.); marcus.hacker@meduniwien.ac.at (M.H.)
2 Department of Pharmaceutical Sciences, Division of Pharmaceutical Chemistry, Faculty of Life Sciences, University of Vienna, 1090 Vienna, Austria; marlon.millard@univie.ac.at (M.M.); a01546417@unet.univie.ac.at (D.F.); a01631177@unet.univie.ac.at (V.M.); wolfgang.holzer@univie.ac.at (W.H.); thierry.langer@univie.ac.at (T.L.)
3 CBmed GmbH—Center for Biomarker Research in Medicine, 8036 Graz, Austria
* Correspondence: verena.pichler@univie.ac.at (V.P.); Tel.: +43-1-4277-55624

Table of Contents

Docking Poses and Pharmacophores 2

Stability in Cell Culture Medium 6

MTT Assay 6

Single-concentration Radioligand Binding Assay 6

HPLC Chromatograms Error! Bookmark not defined.

NMR Spectra 12

Docking Poses and Pharmacophores

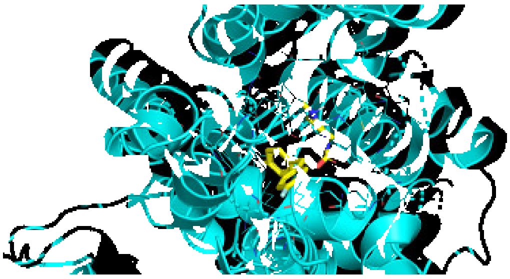
(**a**)

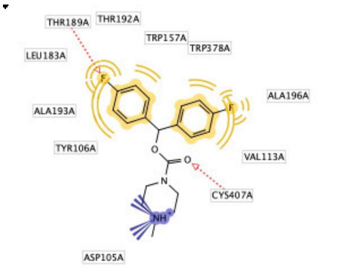

(**b**)

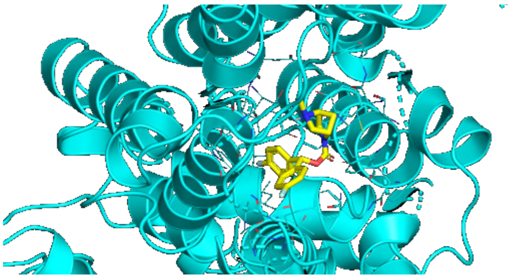
(**c**)

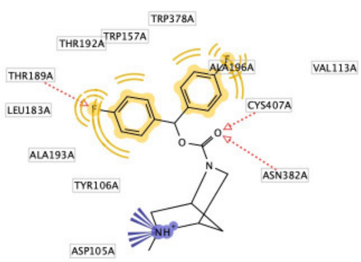

(**d**)

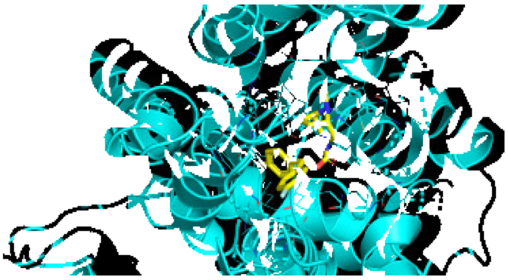
(**e**)

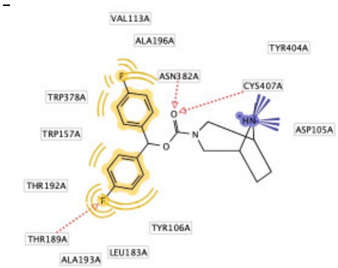

(**f**)

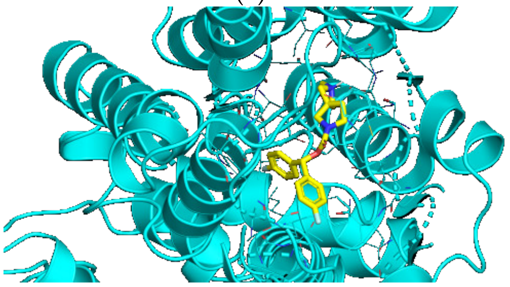
(**g**)

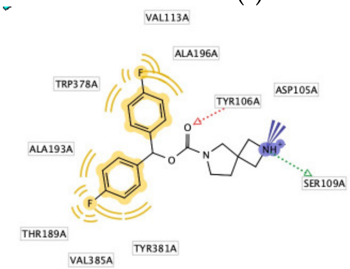

(**h**)

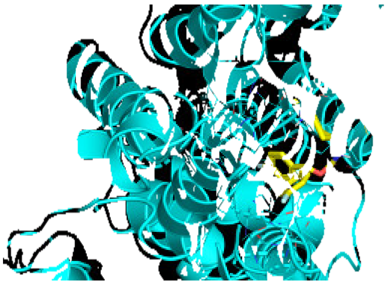
(**i**)

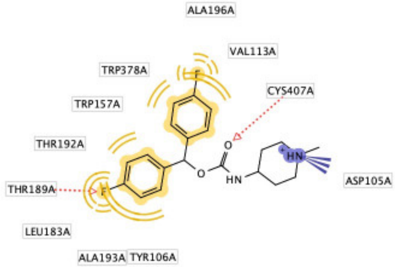

(**j**)

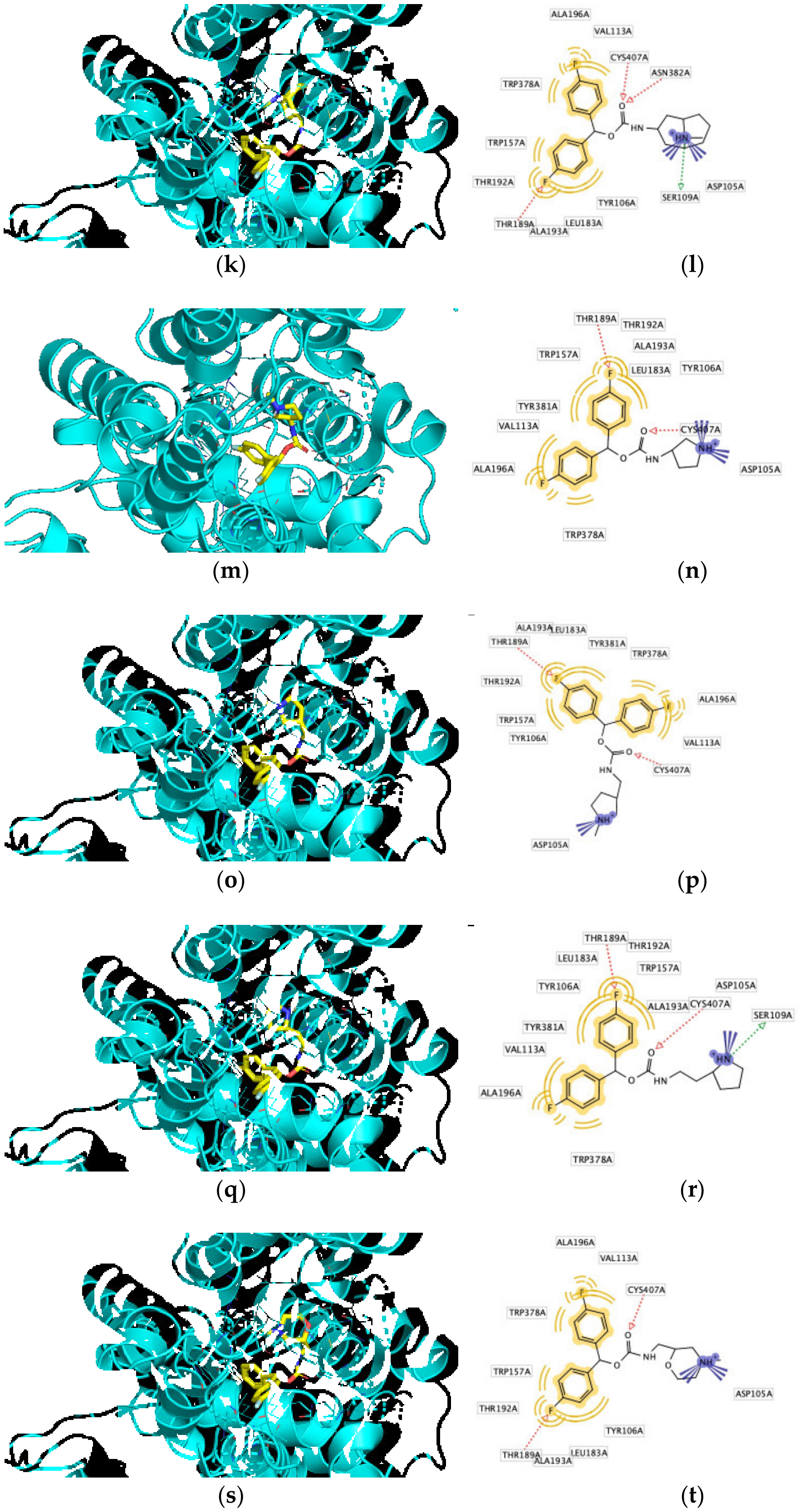

Figure S1. Docking poses for compounds **1**, **3**–**6**, and **8**–**12** (carbons in yellow) in the orthosteric binding site of M_1 (PDB 5CXV) and the corresponding 2D pharmacophores. In case of chiral secondary carbamates, only one enantiomer is shown. (**a**) docking pose of **1**; (**b**) 2D pharmacophore of **1**; (**c**) docking pose of **3**; (**d**) 2D pharmacophore of **3**; (**e**) docking pose of **4**; (**f**) 2D pharmacophore of **4**; (**g**) docking pose of **5**; (**h**) 2D pharmacophore of **5**; (**i**) docking pose of **6**; (**j**) 2D pharmacophore of **6**; (**k**) docking pose of **8**; (**l**) 2D pharmacophore of **8**; (**m**) docking pose of **9**; (**n**) 2D pharmacophore of **9**; (**o**) docking pose

of **10**; (**p**) 2D pharmacophore of **10**; (**q**) docking pose of **11**; (**r**) 2D pharmacophore of **11**; (**s**) docking pose of **12**; (**t**) 2D pharmacophore of **12**.

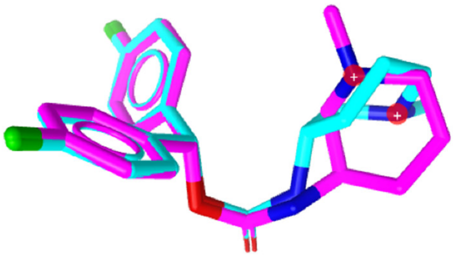

(**a**)

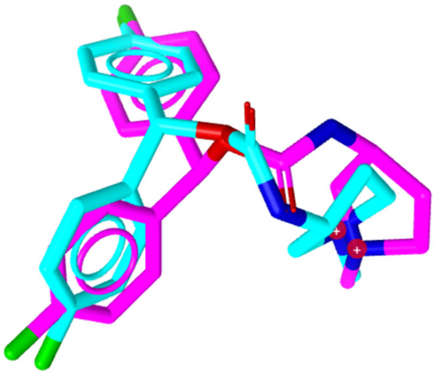

(**b**)

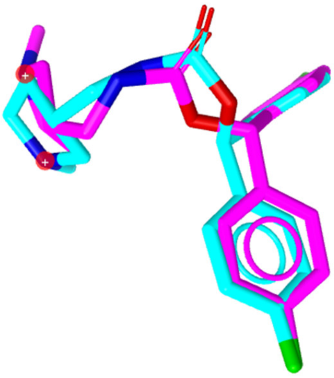

(**c**)

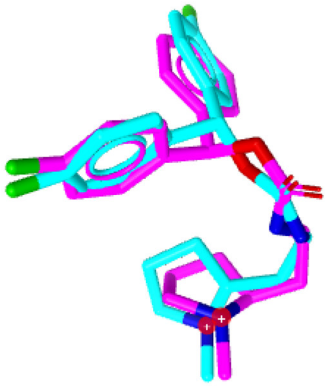

(**d**)

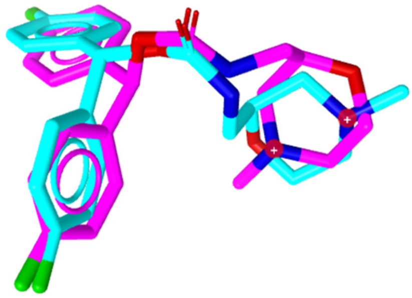

(**e**)

Figure S2. Superimposed docking poses for the enantiomeric pairs of **7** and **9–12**. (**a**) docking poses of (*R*)- and (*S*)-**7**; (**b**) docking poses of (*R*)- and (*S*)-**9**; (**c**) docking poses of (*R*)- and (*S*)-**10**; (**d**) docking poses of (*R*)- and (*S*)-**11**; (**e**) docking poses of (*R*)- and (*S*)-**12.**

Stability in Cell Culture Medium

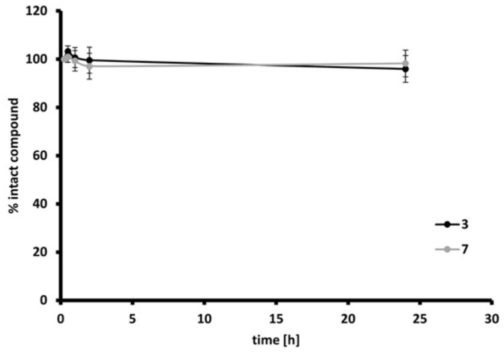

Figure S3. Stability of **3** and **7** in fully supplemented RPMI1640 cell culture medium at 20 °C. Error bars represent the standard deviation.

MTT Assay

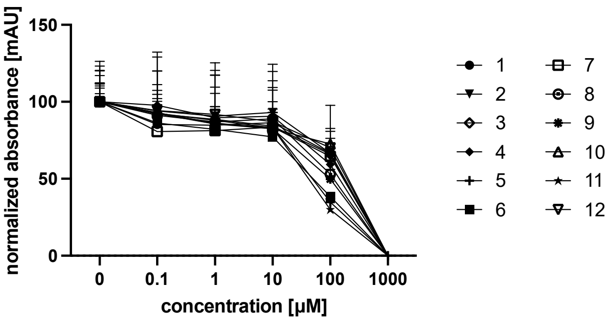

Figure S4. Concentration-dependent Cell viability of **1–12** assessed in living CHO-*h*M_1 cells using an MTT assay. Error bars represent the standard deviation.

Single-concentration Radioligand Binding Assay

Percent displacements of compounds that were excluded from full-range concentration dependent radioligand binding assays to determine their affinities are displayed in Table S1.

Table S1. Percent displacements of [^{3}H]NMS binding on cell membranes derived from CHO-K1 cells expressing *h*Mx receptors at ligand concentrations corresponding to a K_i value of 1 µM according to the Cheng-Prusoff Equation.

	Displacement[1] ± SD (%)				
**Cmpd.**	*h*M_1	*h*M_2	*h*M_3	*h*M_4	*h*M_5
**6**	52 ± 8	10 ± 7	29 ± 5	48 ± 9	54 ± 8
**11**	53 ± 8	37 ± 15	62 ± 3	54 ± 13	59 ± 17
**12**	39 ± 5	8 ± 5	25 ± 9	20 ± 8	43 ± 11

[1] Each value is the mean of at least three independent experiments carried out in triplicate.

Purity measured by HPLC

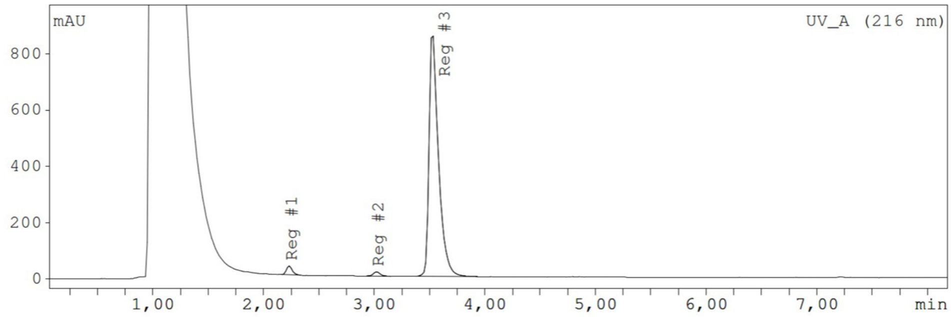

Integration UV_A (216 nm)

Substanz	Ret min	Typ	Fläche mAU*min	%Fläche %
Reg #1	2,23	BB	117,855	2,22
Reg #2	3,02	BB	65,716	1,24
Reg #3	3,53	BB	5119,570	96,54
Summe in ROI			5303,142	100,00

Figure S5. Isocratic HPLC chromatogram of **1**. 60% ACN in 25 mM $NH_4H_2PO_4$ buffer pH 9.3.

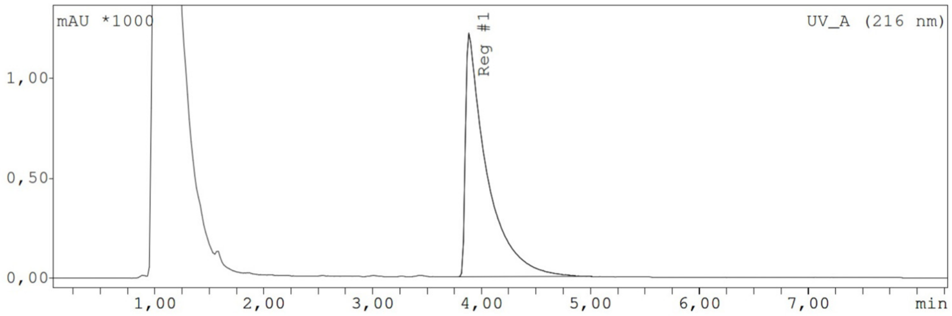

Integration UV_A (216 nm)

Substanz	Ret min	Typ	Fläche mAU*min	%Fläche %
Reg #1	3,90	BB	16511,94	100,00
Summe in ROI			16511,94	100,00

Figure S6. Isocratic HPLC chromatogram of **2**. 60% ACN in 25 mM $NH_4H_2PO_4$ buffer pH 9.3.

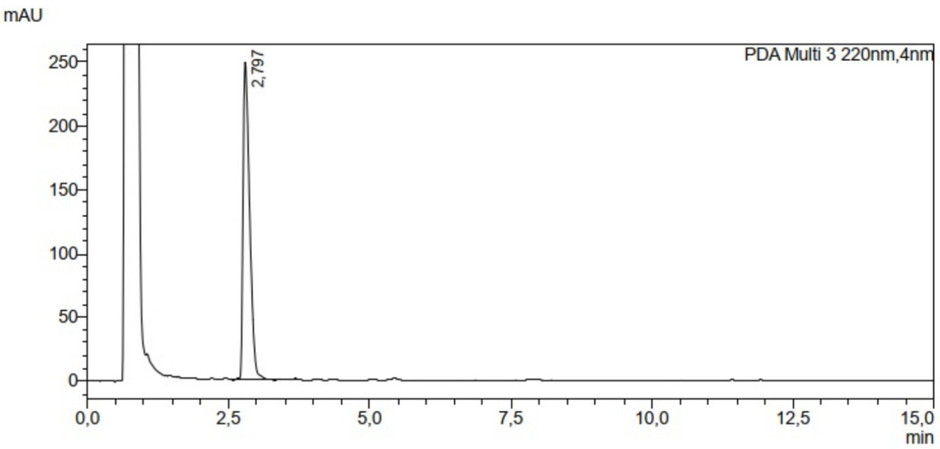

<Peak Table>

PDA Ch3 220nm

Peak#	Ret. Time	Area	Height	Area%	Conc.	Mark
1	2,797	2023938	248995	100,000	0,000	M
Total		2023938	248995	100,000		

Figure S7. Isocratic HPLC chromatogram of **3**. 35% ACN in H_2O, 0.1% TFA.

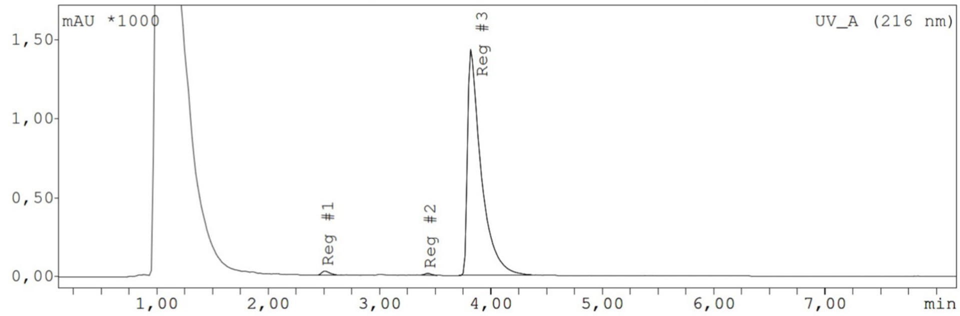

Integration UV_A (216 nm)

Substanz	Ret min	Typ	Fläche mAU*min	%Fläche %
Reg #1	2,52	BB	119,10	0,97
Reg #2	3,43	BB	45,92	0,37
Reg #3	3,83	BB	12171,36	98,66
Summe in ROI			12336,38	100,00

Figure S8. Isocratic HPLC chromatogram of **4**. 60% ACN in 25 mM $NH_4H_2PO_4$ buffer pH 9.3.

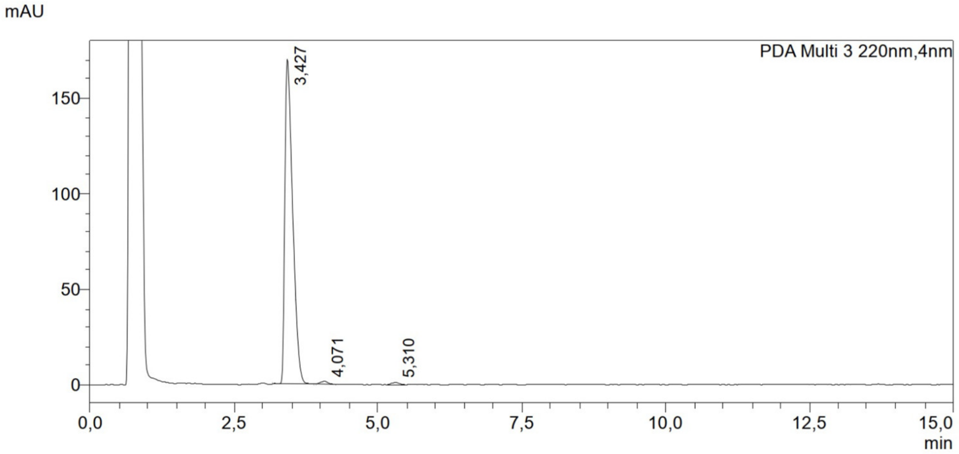

<Peak Table>

PDA Ch3 220nm

Peak#	Ret. Time	Area	Height	Area%	Conc.	Mark
1	3,427	1603220	169659	98,487	0,000	M
2	4,071	12895	1441	0,792	0,000	M
3	5,310	11733	1219	0,721	0,000	M
Total		1627848	172319	100,000		

Figure S9. Isocratic HPLC chromatogram of **5**. 35% ACN in H_2O, 0.1% TFA.

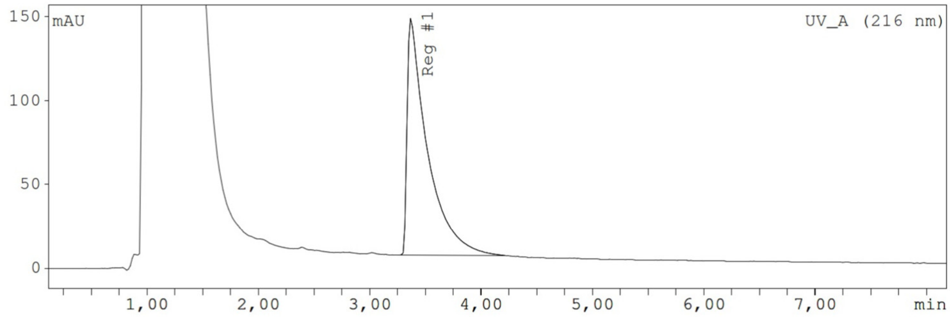

Integration UV_A (216 nm)

Substanz	Ret min	Typ	Fläche mAU*min	%Fläche %
Reg #1	3,38	BB	1783,137	100,00
Summe in ROI			1783,137	100,00

Figure S10. Isocratic HPLC chromatogram of **6**. 60% ACN in 25 mM $NH_4H_2PO_4$ buffer pH 9.3.

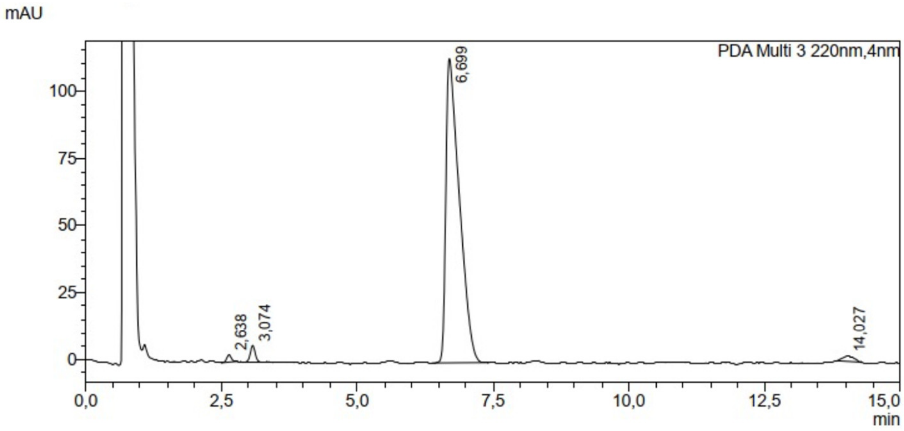

<Peak Table>

PDA Ch3 220nm

Peak#	Ret. Time	Area	Height	Area%	Conc.	Mark
1	2,638	16204	2704	0,791	0,000	M
2	3,074	35634	6092	1,740	0,000	M
3	6,699	1967263	113431	96,036	0,000	M
4	14,027	29372	1917	1,434	0,000	M
Total		2048472	124143	100,000		

Figure S11. Isocratic HPLC chromatogram of **7**. 30% ACN in H_2O, 0.1% TFA.

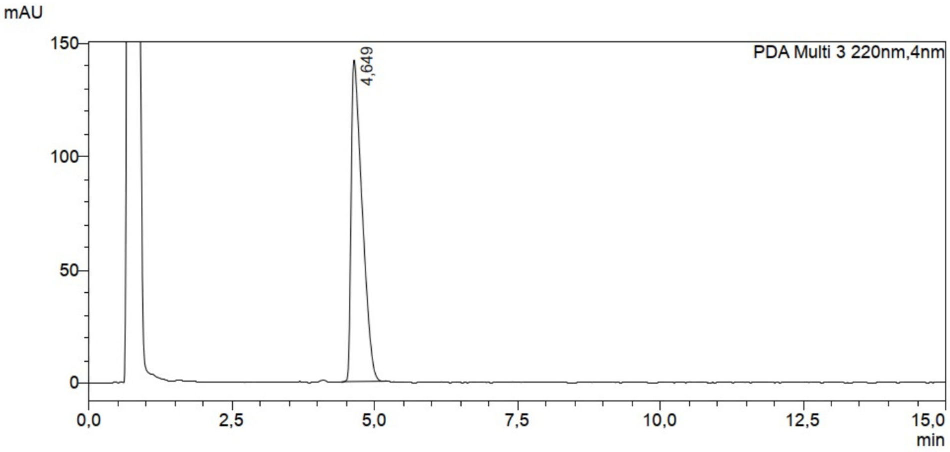

<Peak Table>

PDA Ch3 220nm

Peak#	Ret. Time	Area	Height	Area%	Conc.	Mark
1	4,649	1903512	142075	100,000	0,000	M
Total		1903512	142075	100,000		

Figure S12. Isocratic HPLC chromatogram of **8**. 35% ACN in H_2O, 0.1% TFA.

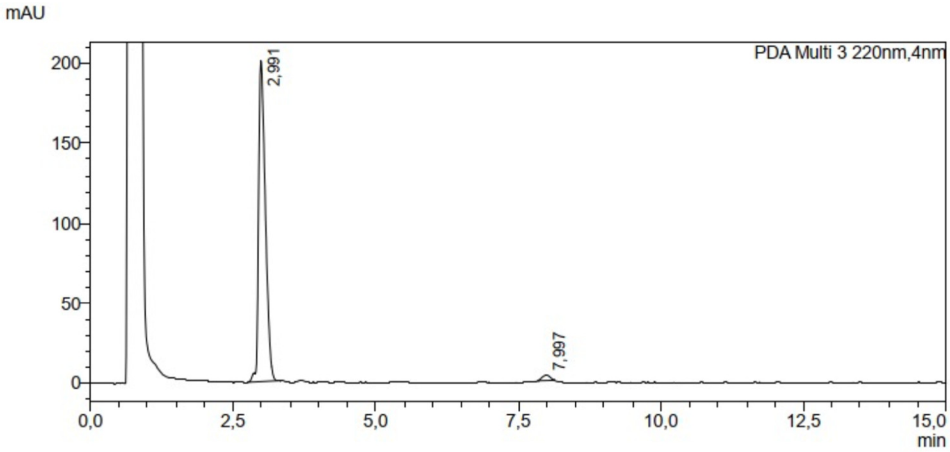

<Peak Table>

PDA Ch3 220nm

Peak#	Ret. Time	Area	Height	Area%	Conc.	Mark
1	2,991	1658055	200594	98,079	0,000	M
2	7,997	32479	3252	1,921	0,000	M
Total		1690535	203847	100,000		

Figure S13. Isocratic HPLC chromatogram of **9**. 35% ACN in H_2O, 0.1% TFA.

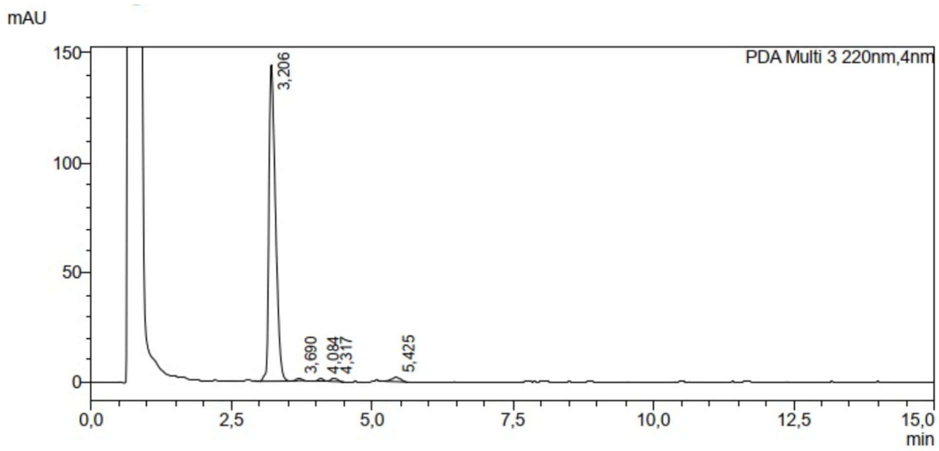

<Peak Table>

PDA Ch3 220nm

Peak#	Ret. Time	Area	Height	Area%	Conc.	Mark
1	3,206	1173117	144193	96,221	0,000	M
2	3,690	5953	900	0,488	0,000	M
3	4,084	8055	1301	0,661	0,000	M
4	4,317	10883	1447	0,893	0,000	M
5	5,425	21185	1896	1,738	0,000	M
Total		1219194	149737	100,000		

Figure S14. Isocratic HPLC chromatogram of **10**. 35% ACN in H_2O, 0.1% TFA.

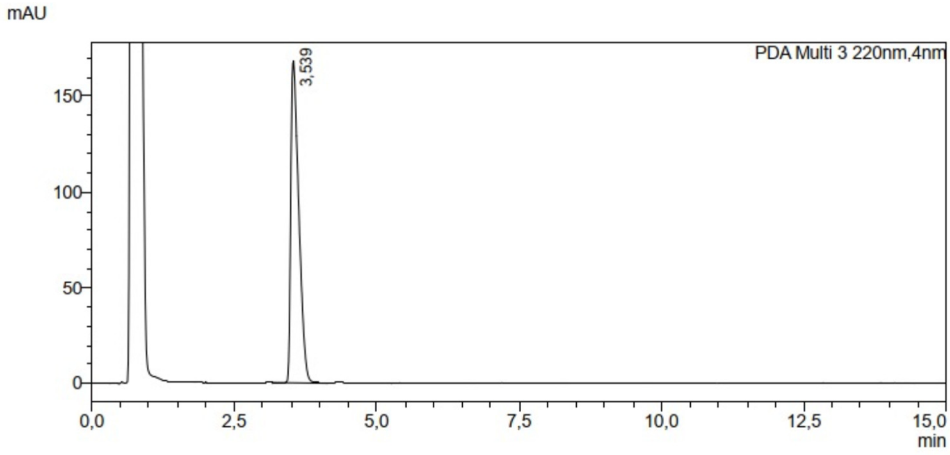

<Peak Table>

PDA Ch3 220nm

Peak#	Ret. Time	Area	Height	Area%	Conc.	Mark
1	3,539	1642714	168041	100,000	0,000	M
Total		1642714	168041	100,000		

Figure S15. Isocratic HPLC chromatogram of **11**. 35% ACN in H_2O, 0.1% TFA.

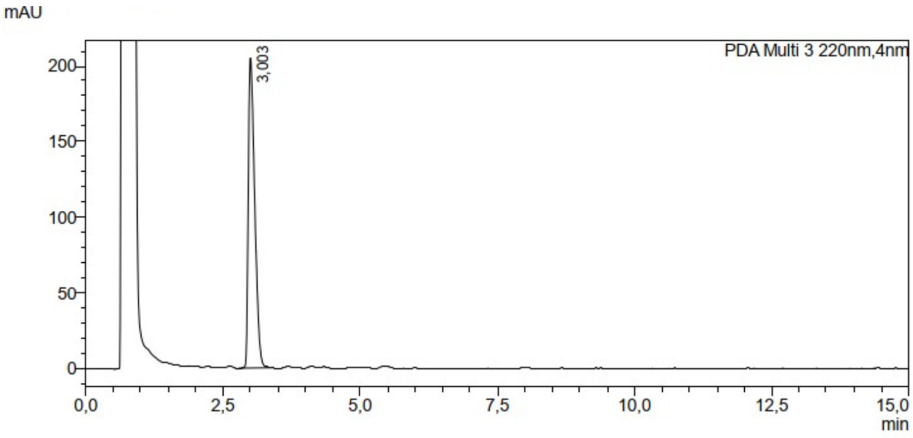

<Peak Table>

PDA Ch3 220nm

Peak#	Ret. Time	Area	Height	Area%	Conc.	Mark
1	3,003	1667422	204334	100,000	0,000	M
Total		1667422	204334	100,000		

Figure S16. Isocratic HPLC chromatogram of **12**. 35% ACN in H_2O, 0.1% TFA.

NMR Spectra

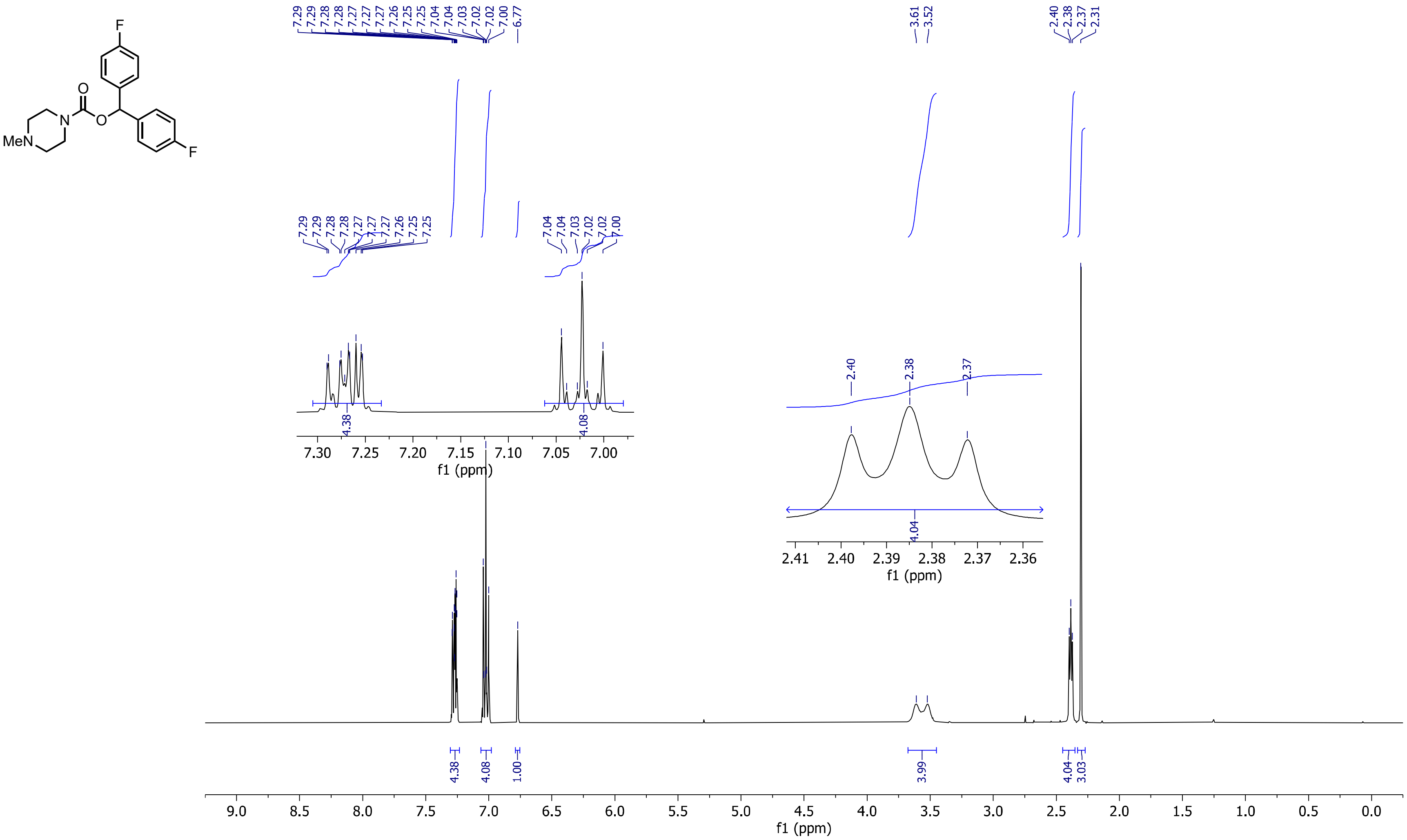

Figure S17. ^{1}H NMR spectrum of **1** (400 MHz, $CDCl_3$).

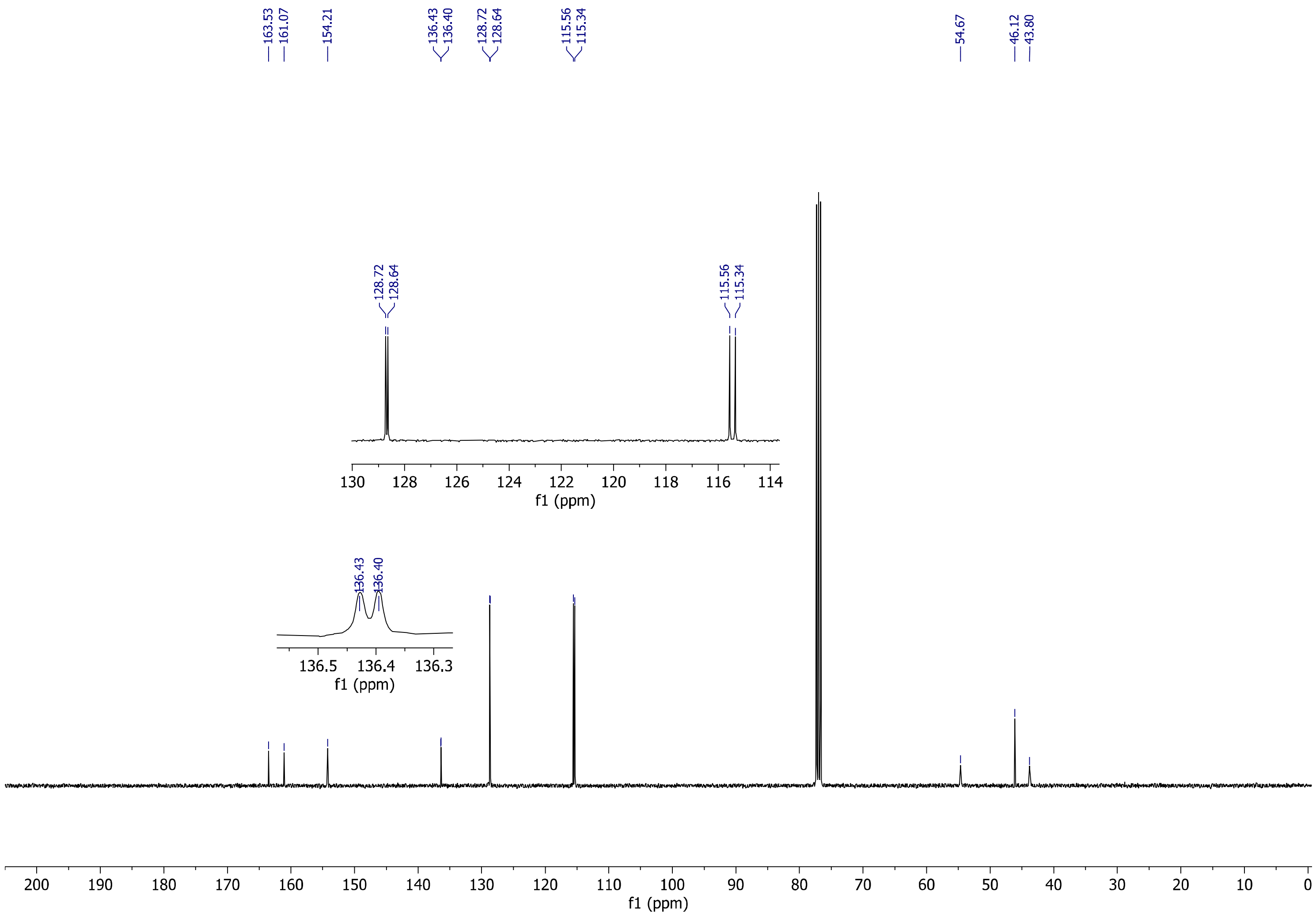

Figure S18. ^{13}C NMR spectrum of **1** (100 MHz, $CDCl_3$).

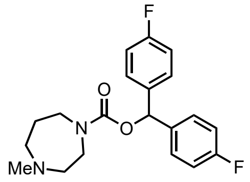

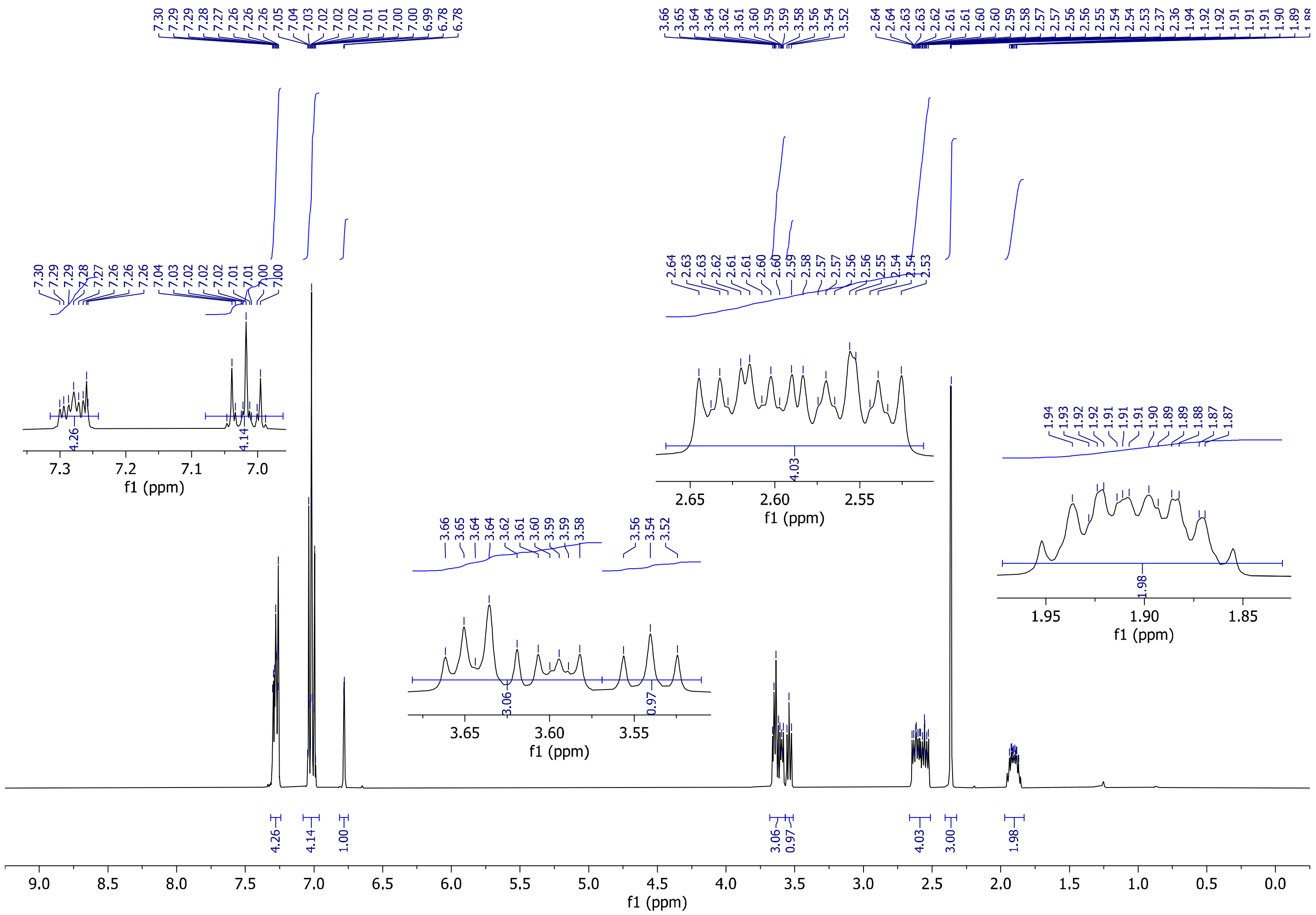

Figure S19. ^{1}H NMR spectrum of **2** (400 MHz, $CDCl_3$).

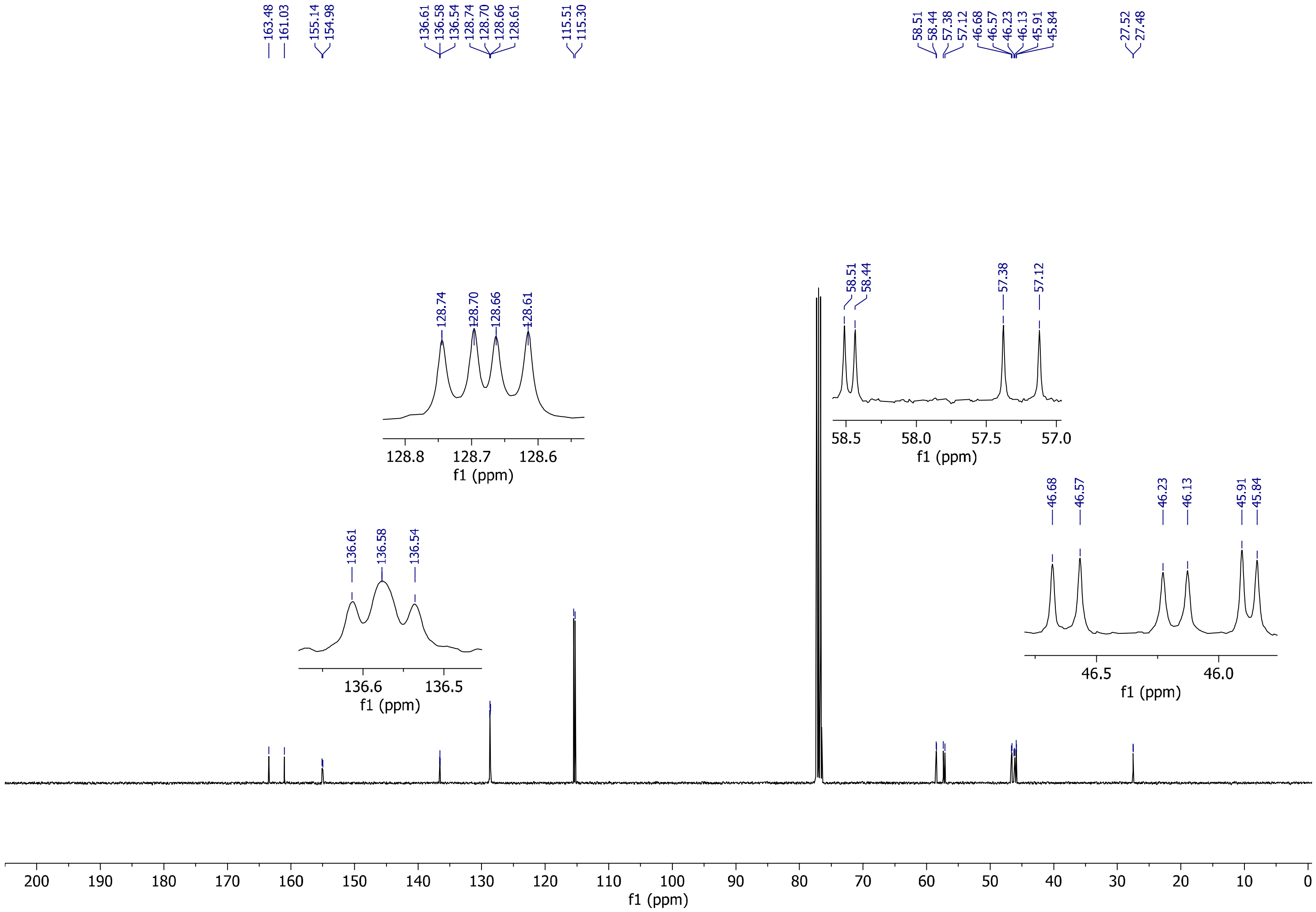

Figure S20. ^{13}C NMR spectrum of **2** (100 MHz, $CDCl_3$).

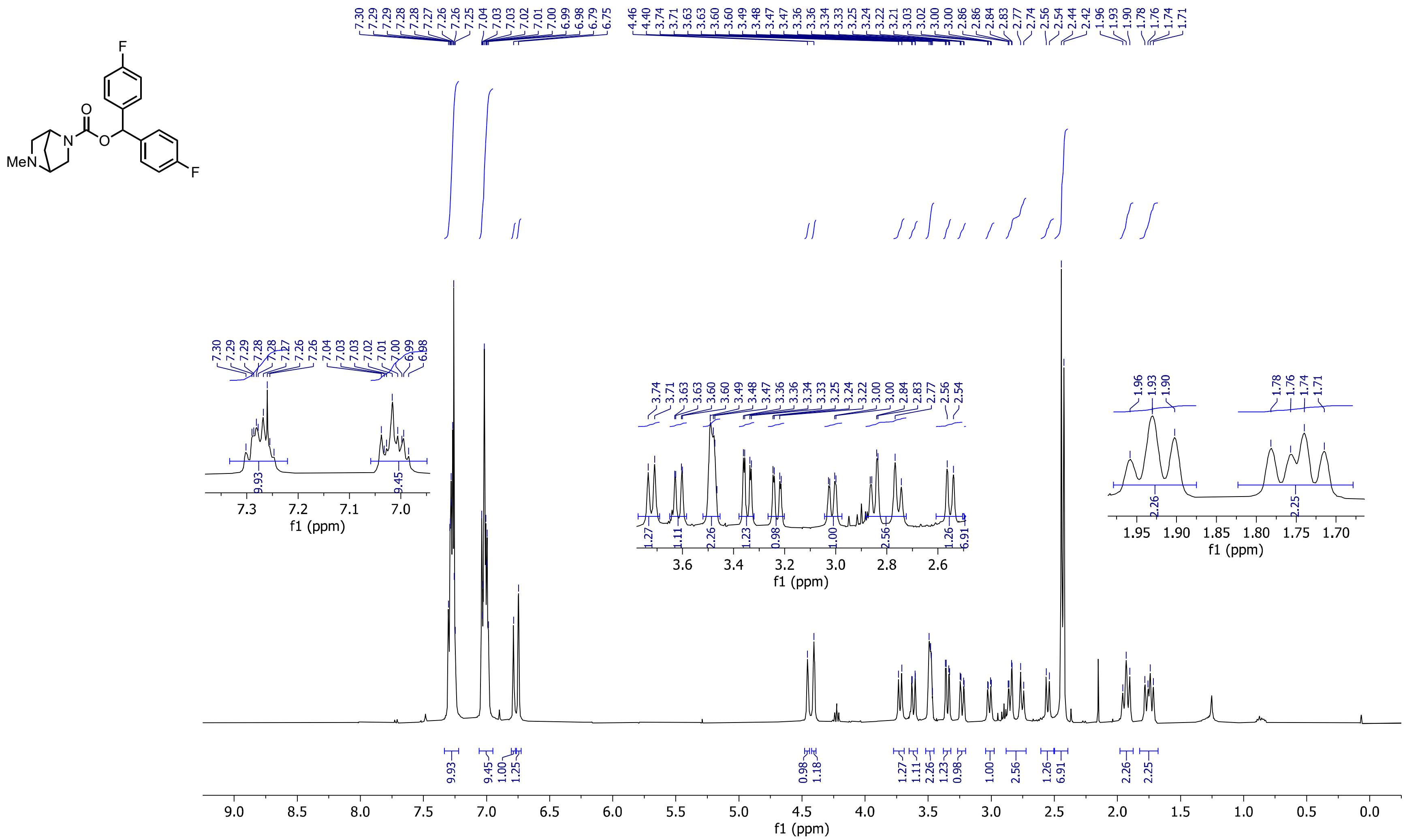

Figure S21. ^{1}H NMR spectrum of **3** (400 MHz, $CDCl_3$).

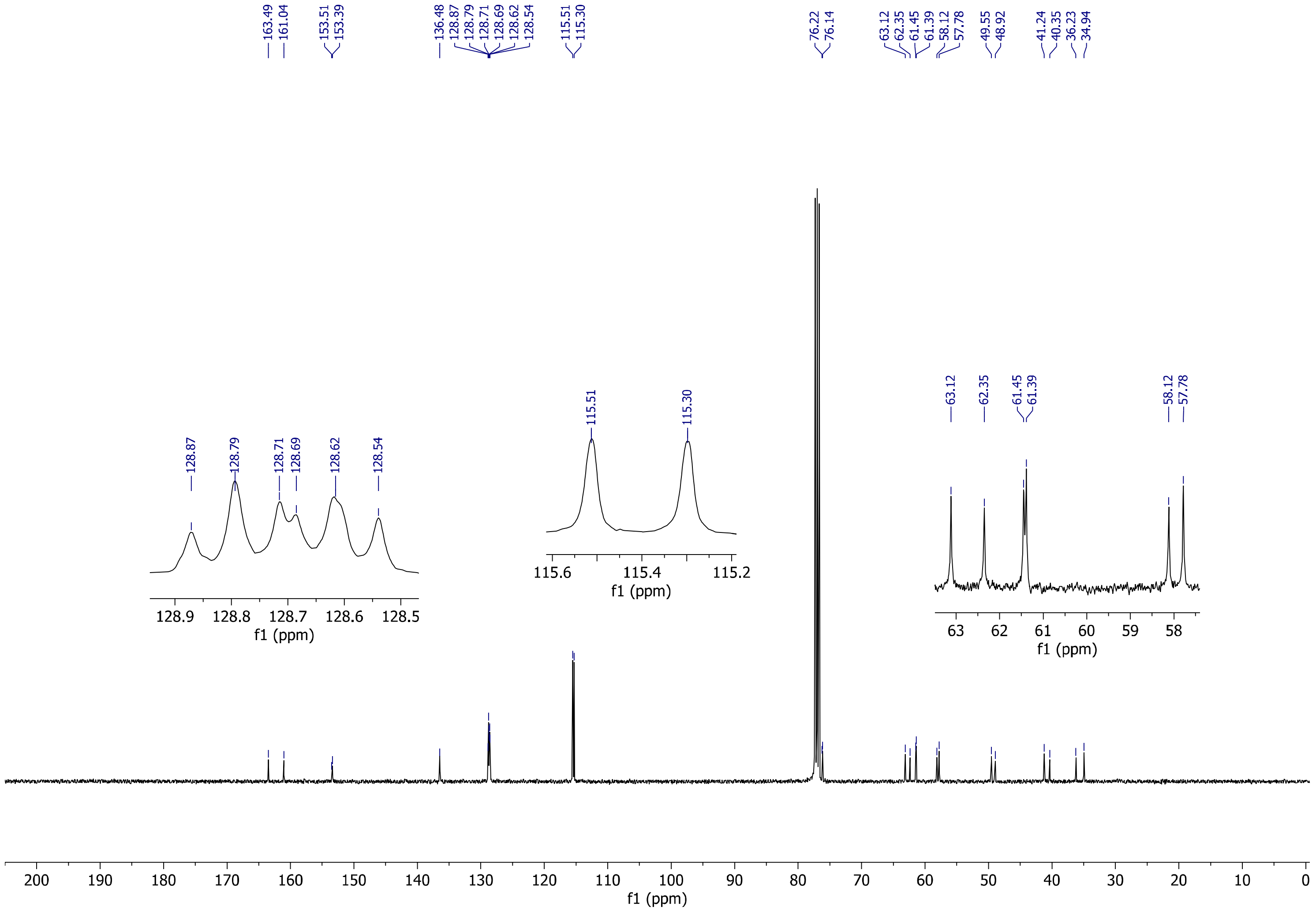

Figure S22. ^{13}C NMR spectrum of **3** (100 MHz, $CDCl_3$).

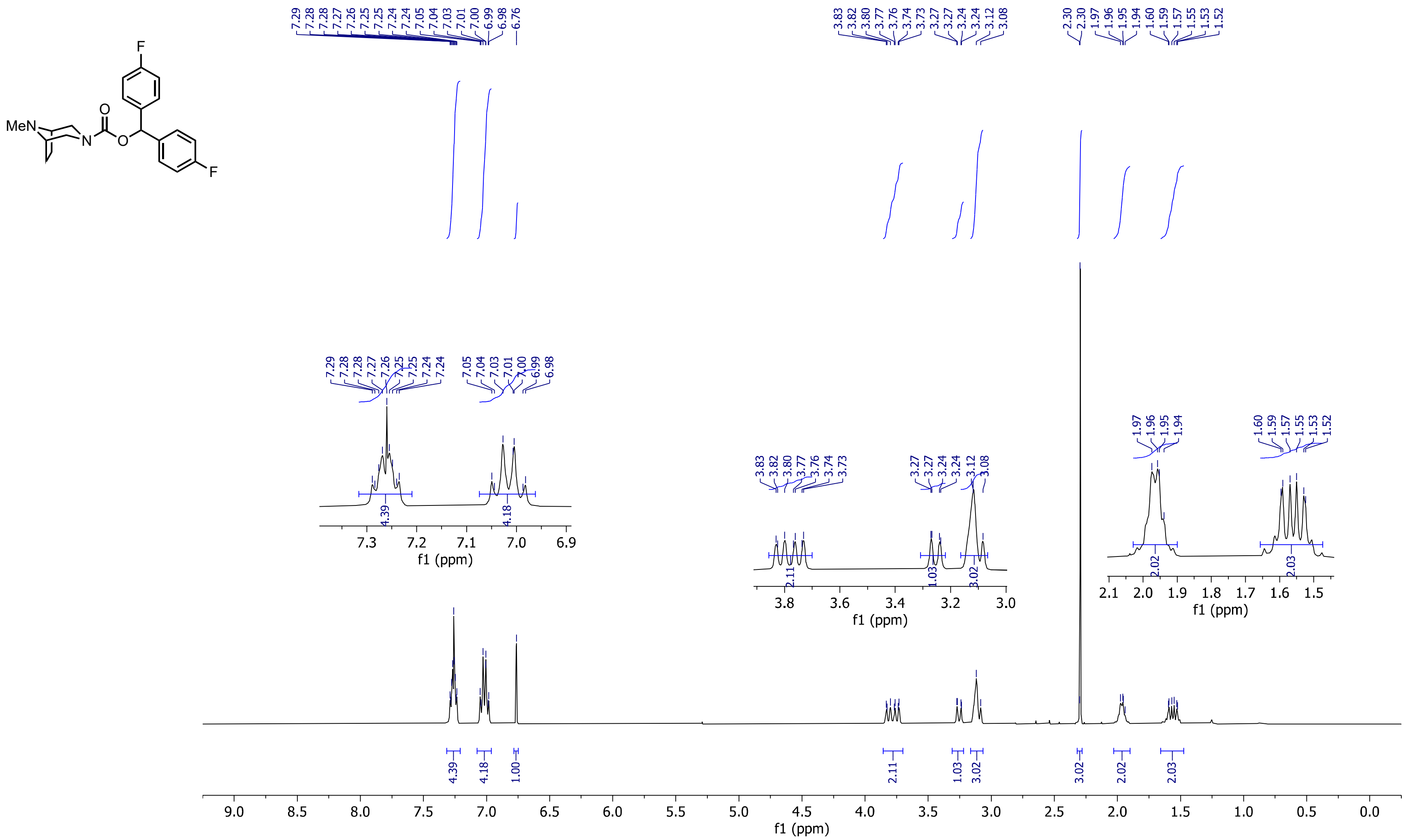

Figure S23. ^{1}H NMR spectrum of **4** (400 MHz, $CDCl_3$).

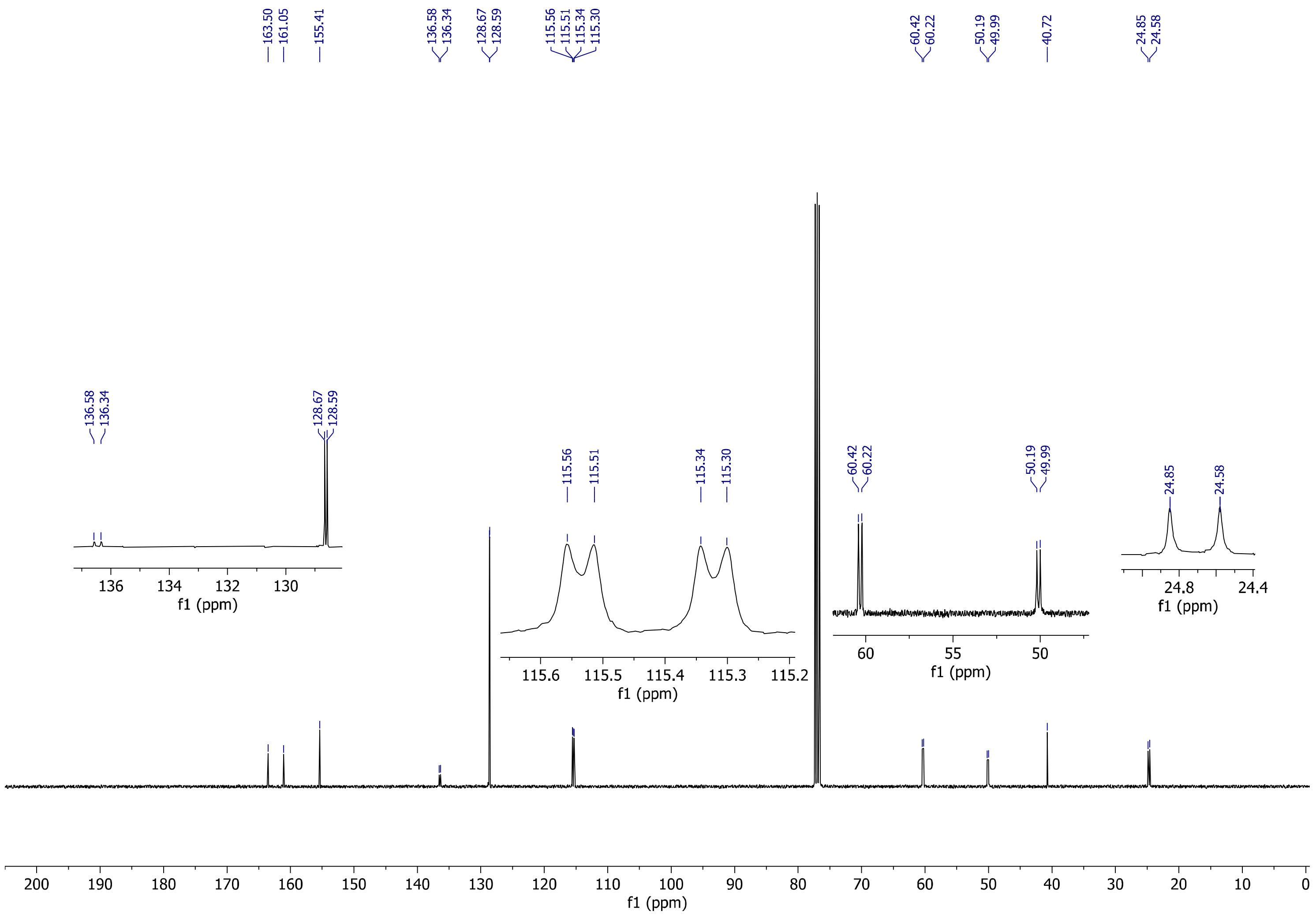

Figure S24. ^{13}C NMR spectrum of **4** (100 MHz, $CDCl_3$).

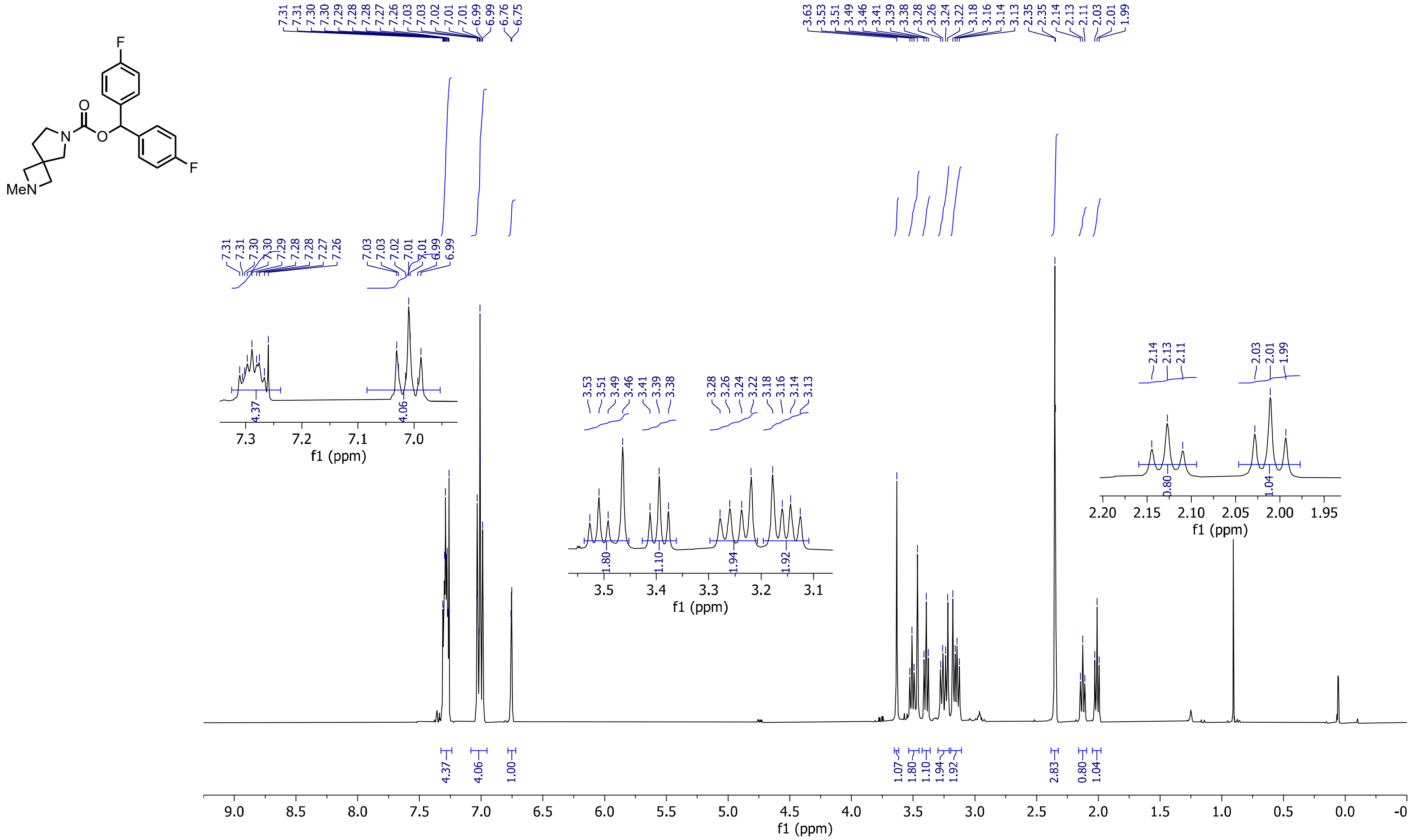

Figure S25. ^{1}H NMR spectrum of **5** (400 MHz, $CDCl_3$).

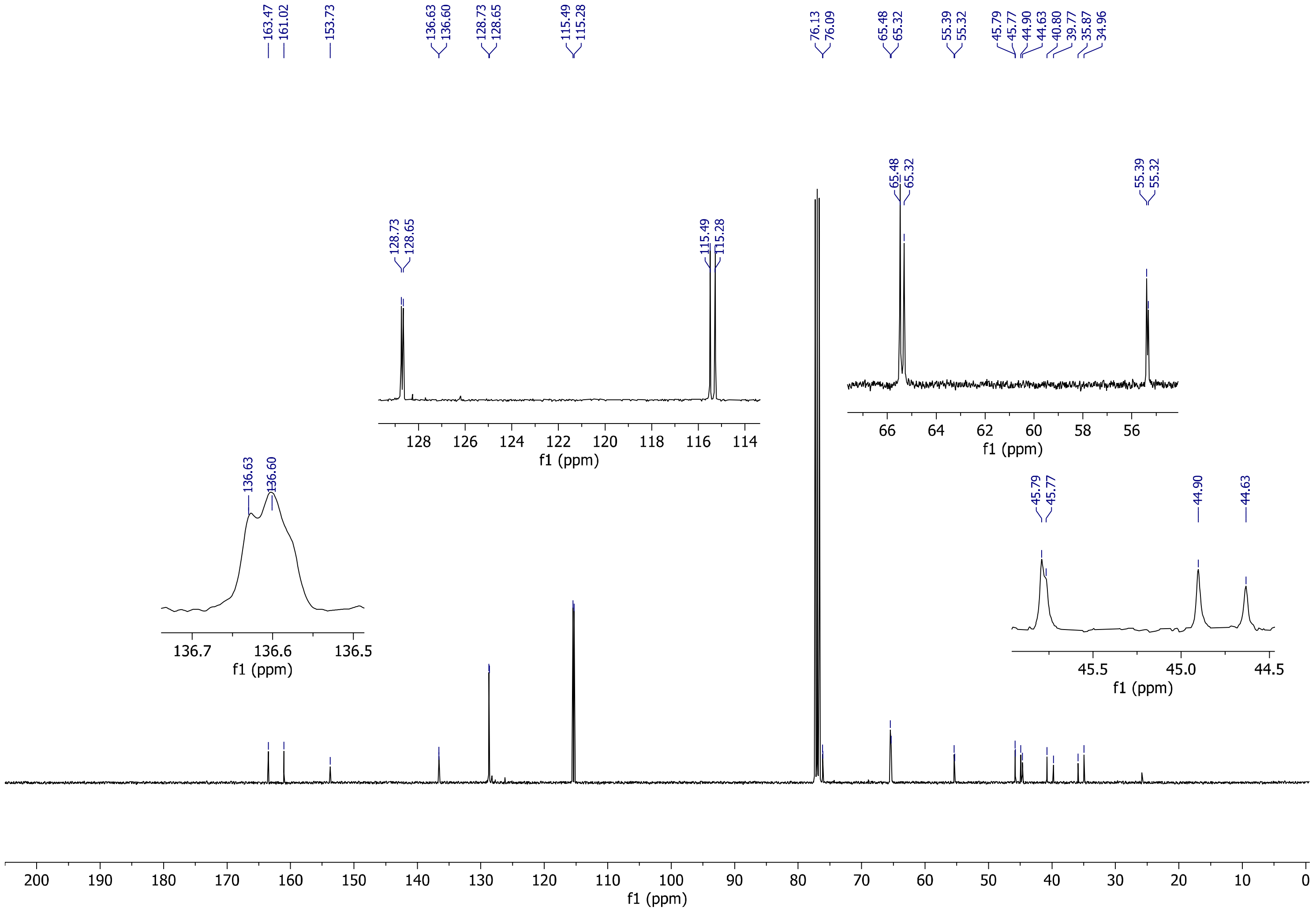

Figure S26. ^{13}C NMR spectrum of **5** (100 MHz, $CDCl_3$).

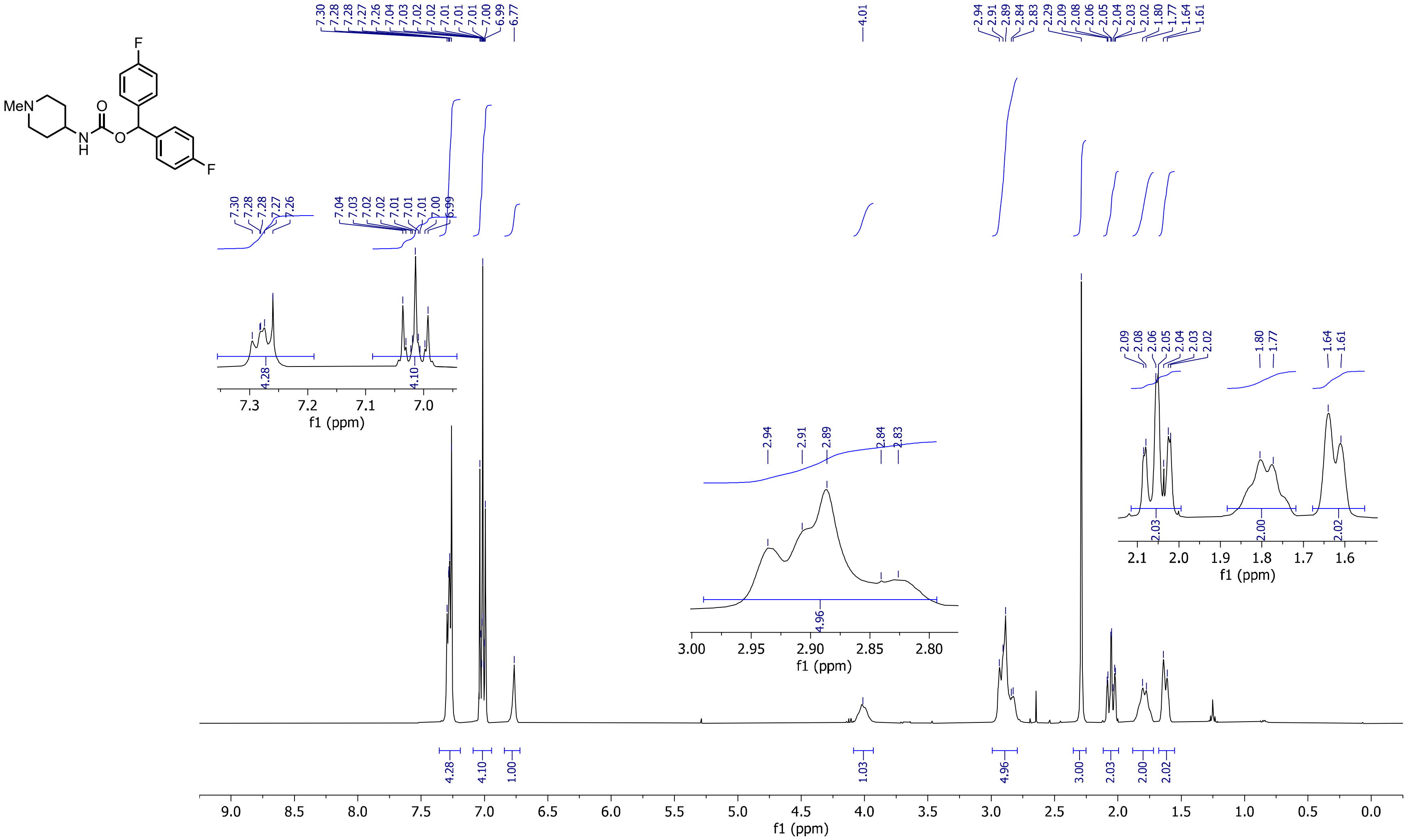

Figure S27. ^{1}H NMR spectrum of **6** (400 MHz, $CDCl_3$).

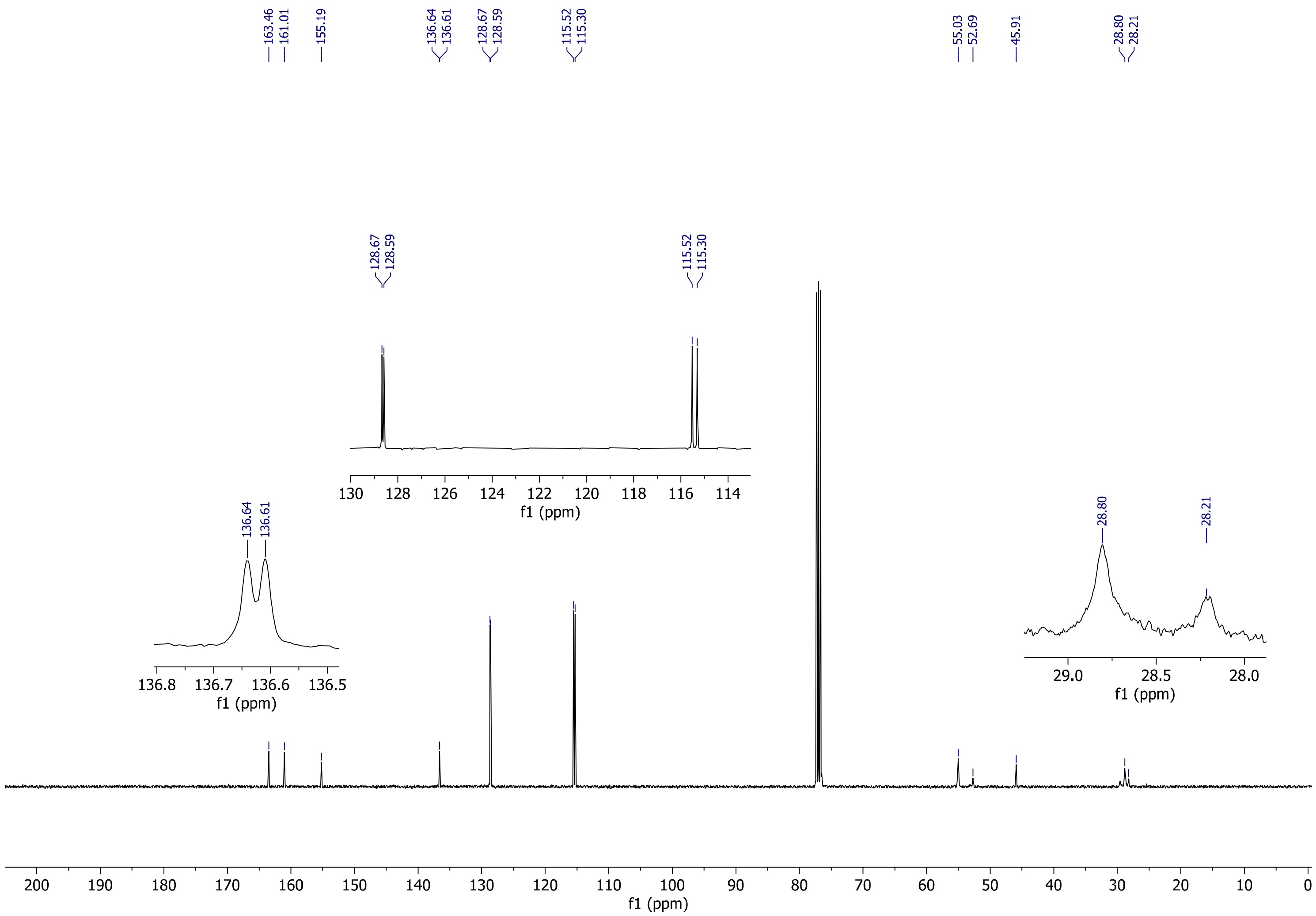

Figure S28. ^{13}C NMR spectrum of **6** (100 MHz, $CDCl_3$).

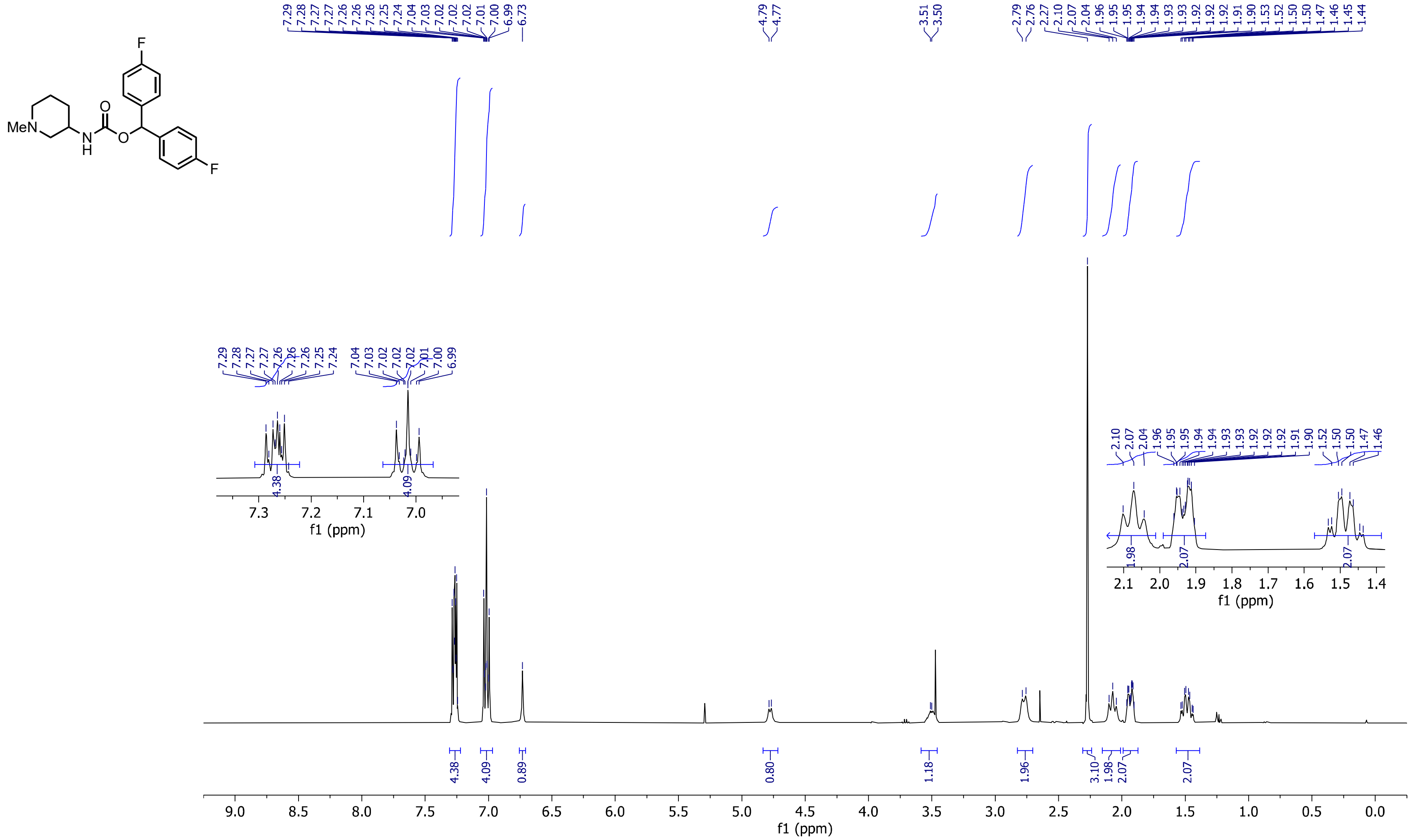

Figure S29. ^{1}H NMR spectrum of **7** (400 MHz, $CDCl_3$).

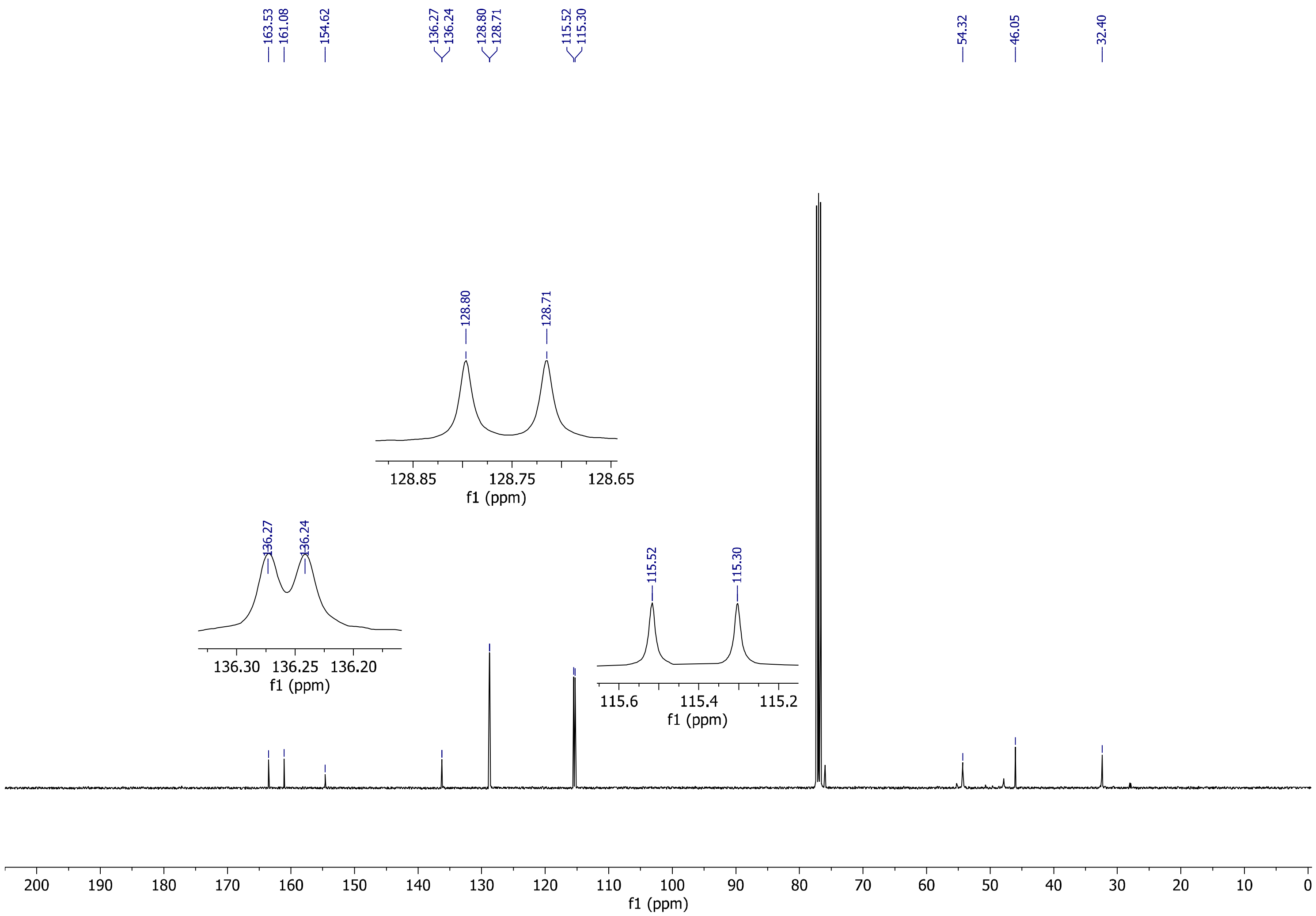

Figure S30. ^{13}C NMR spectrum of **7** (100 MHz, $CDCl_3$).

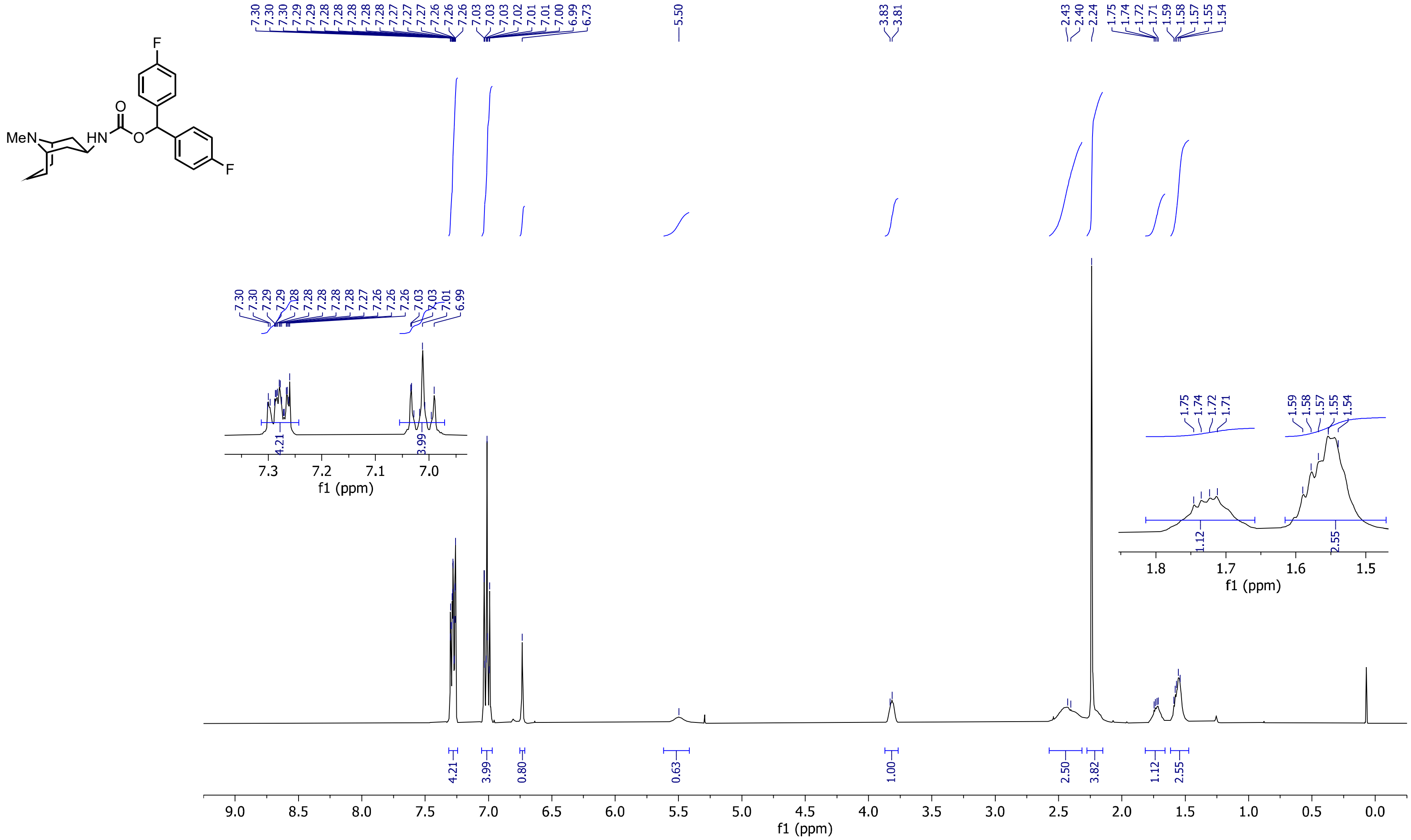

Figure S31. ^{1}H NMR spectrum of **8** (400 MHz, $CDCl_3$).

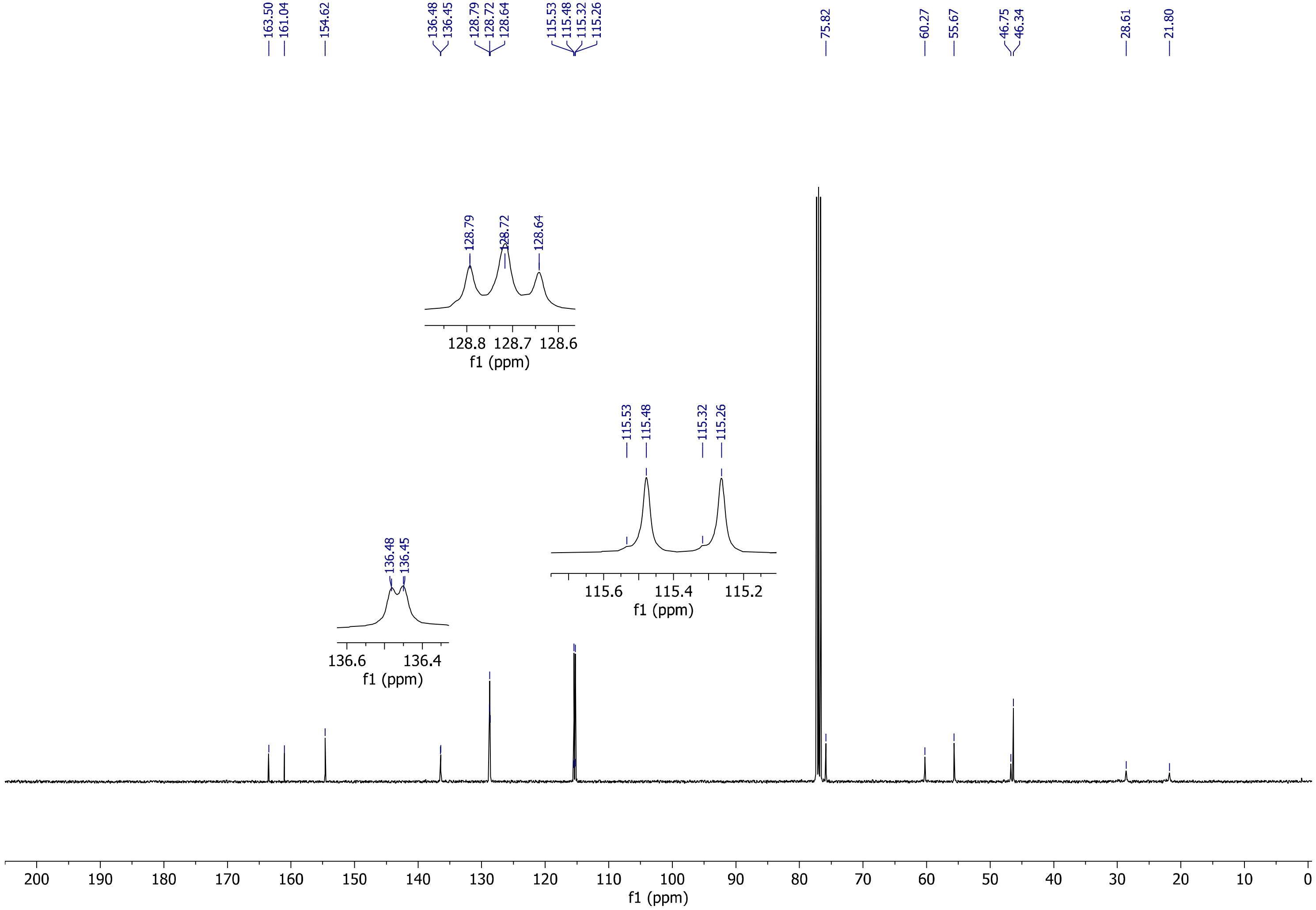

Figure S32. ^{13}C NMR spectrum of **8** (100 MHz, $CDCl_3$).

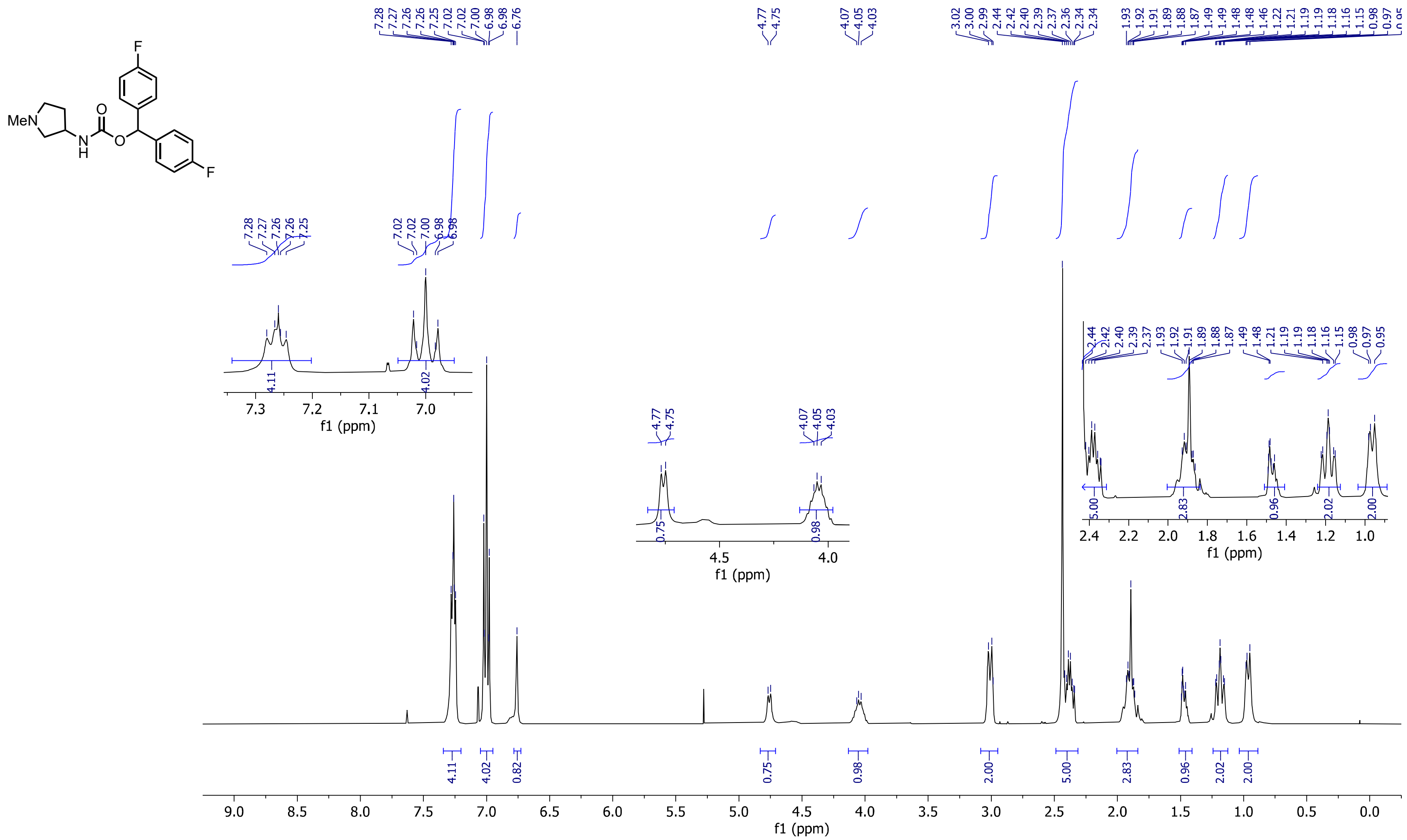

Figure S33. ^{1}H NMR spectrum of **9** (400 MHz, $CDCl_3$).

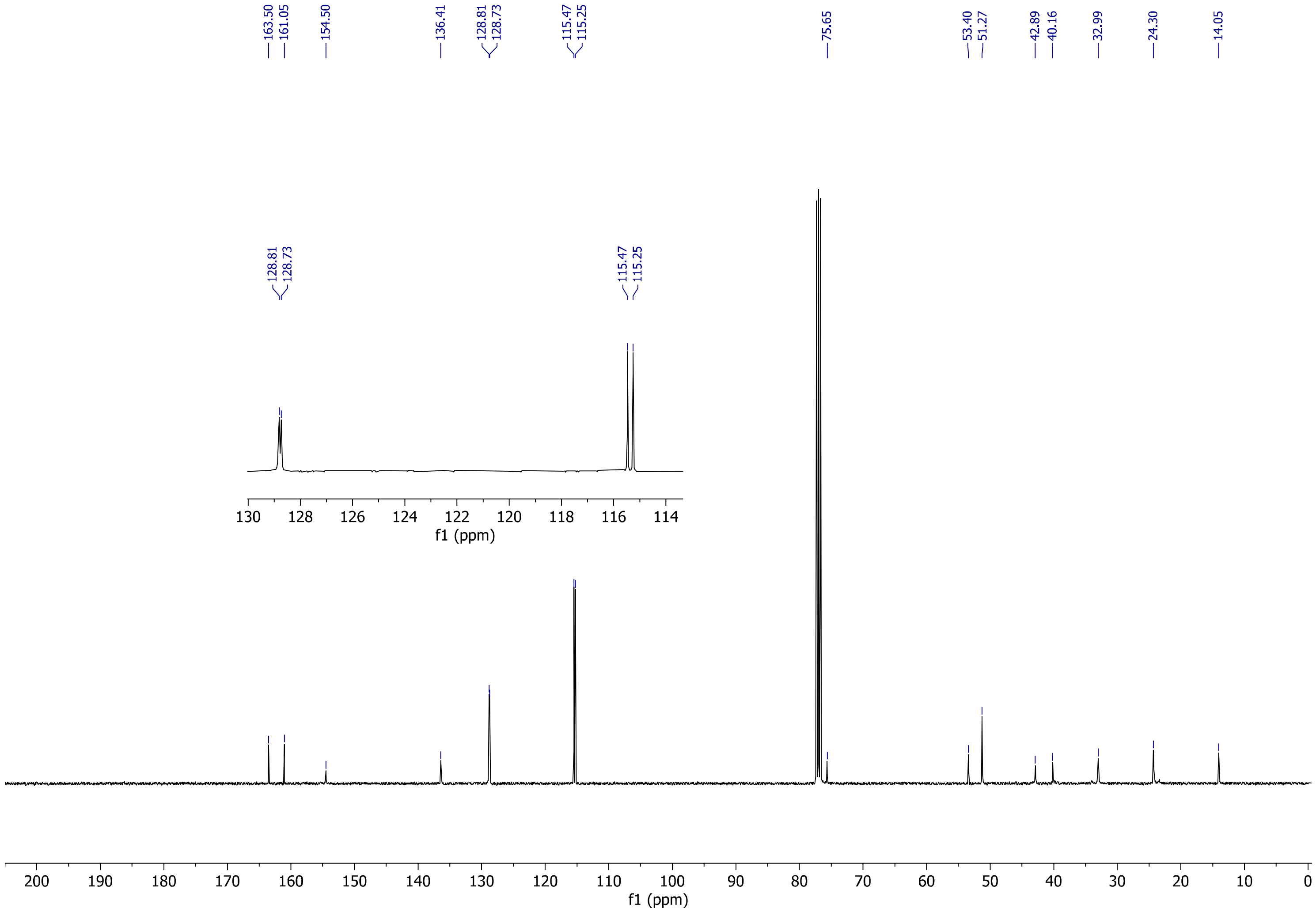

Figure S34. ^{13}C NMR spectrum of **9** (100 MHz, $CDCl_3$).

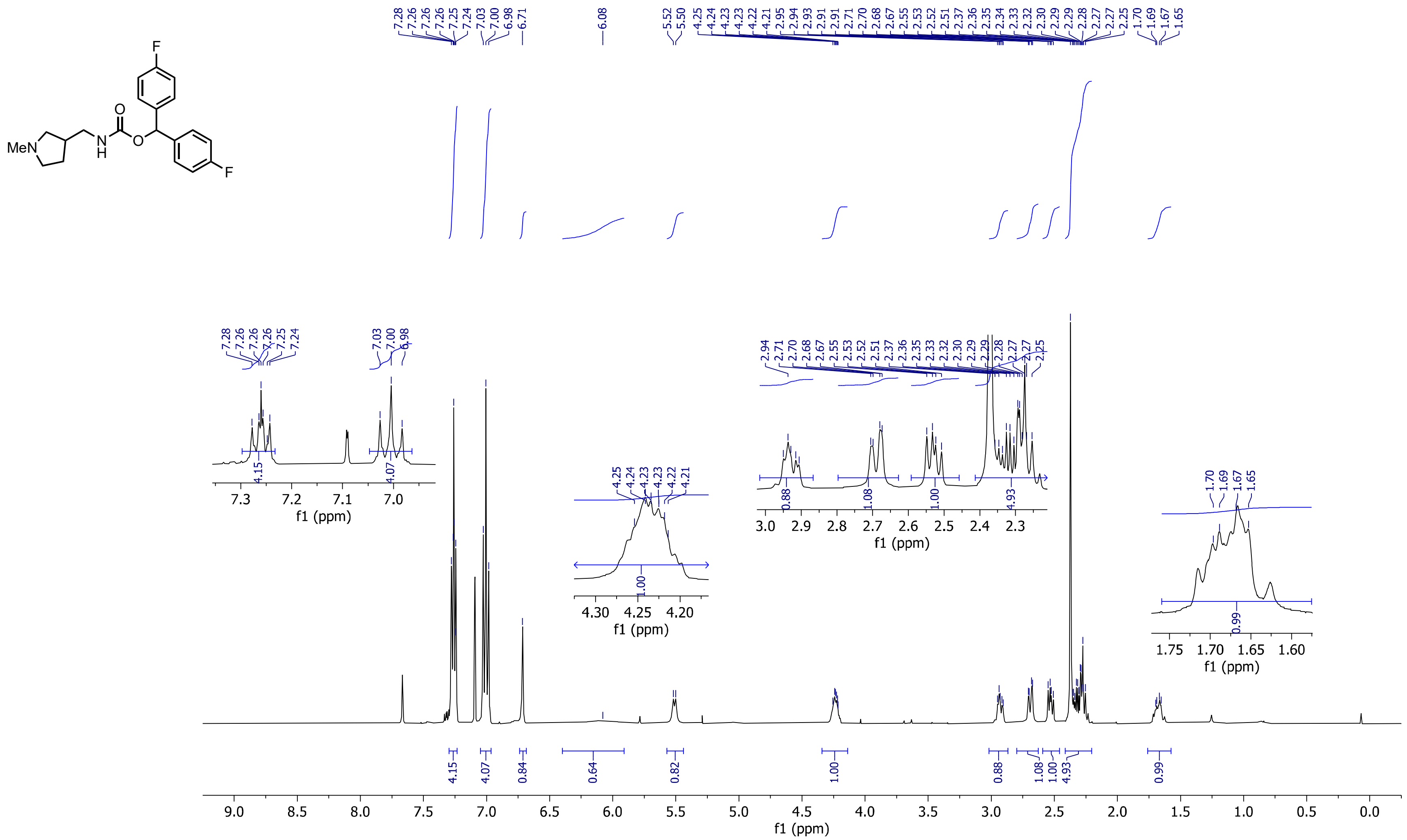

Figure S35. ^{1}H NMR spectrum of **10** (400 MHz, $CDCl_3$).

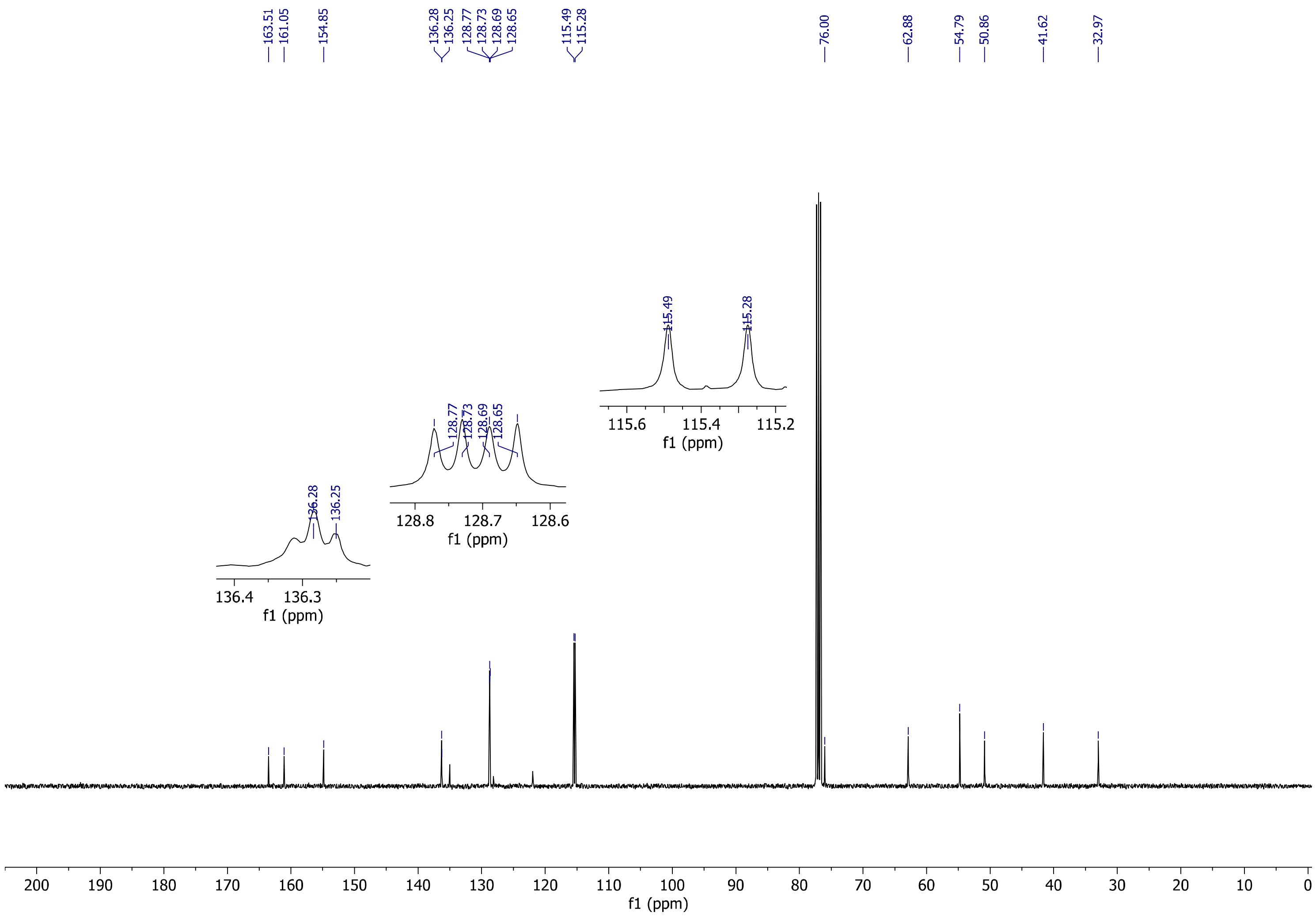

Figure S36. ^{13}C NMR spectrum of **10** (100 MHz, $CDCl_3$).

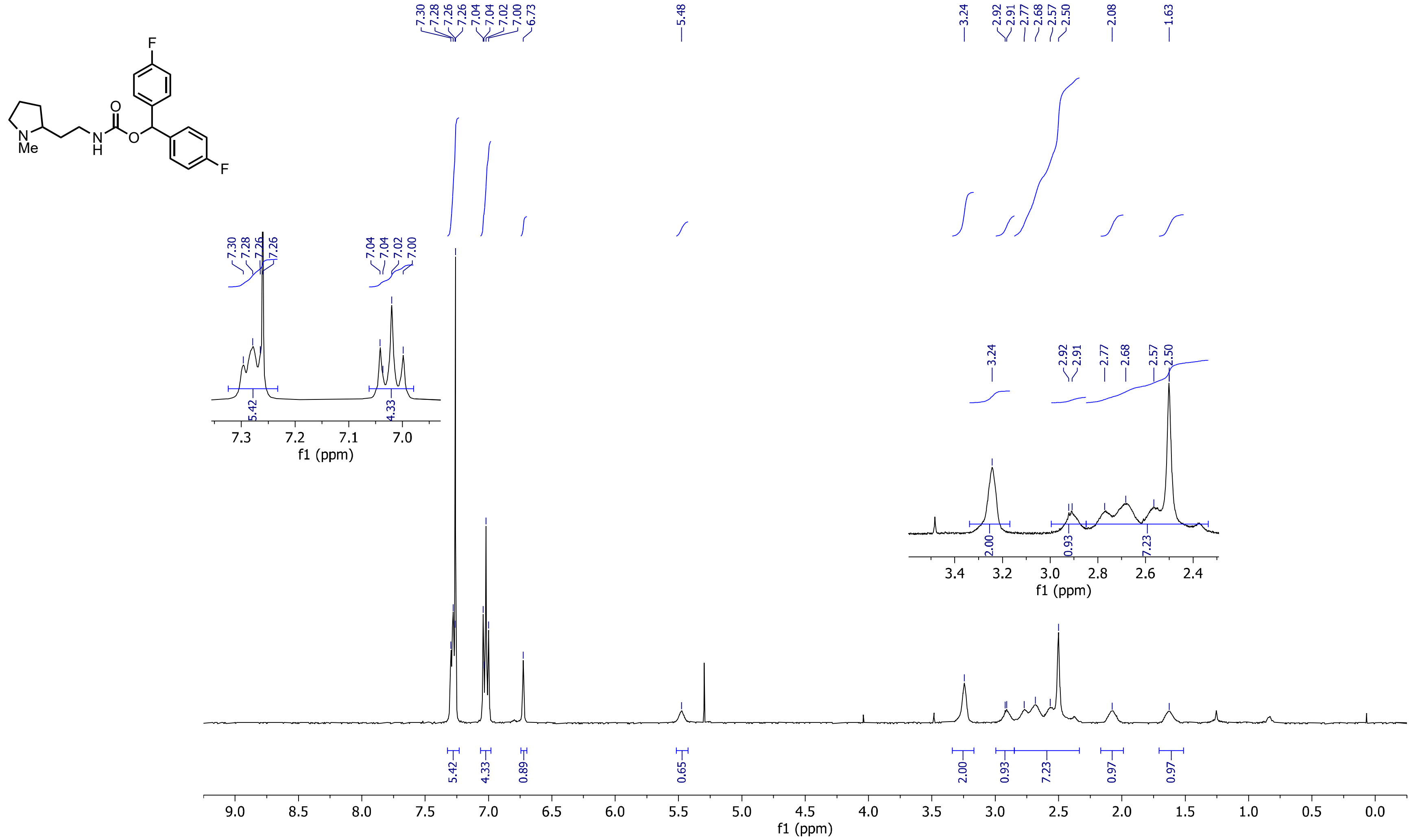

Figure S37. ^{1}H NMR spectrum of **11** (400 MHz, $CDCl_3$).

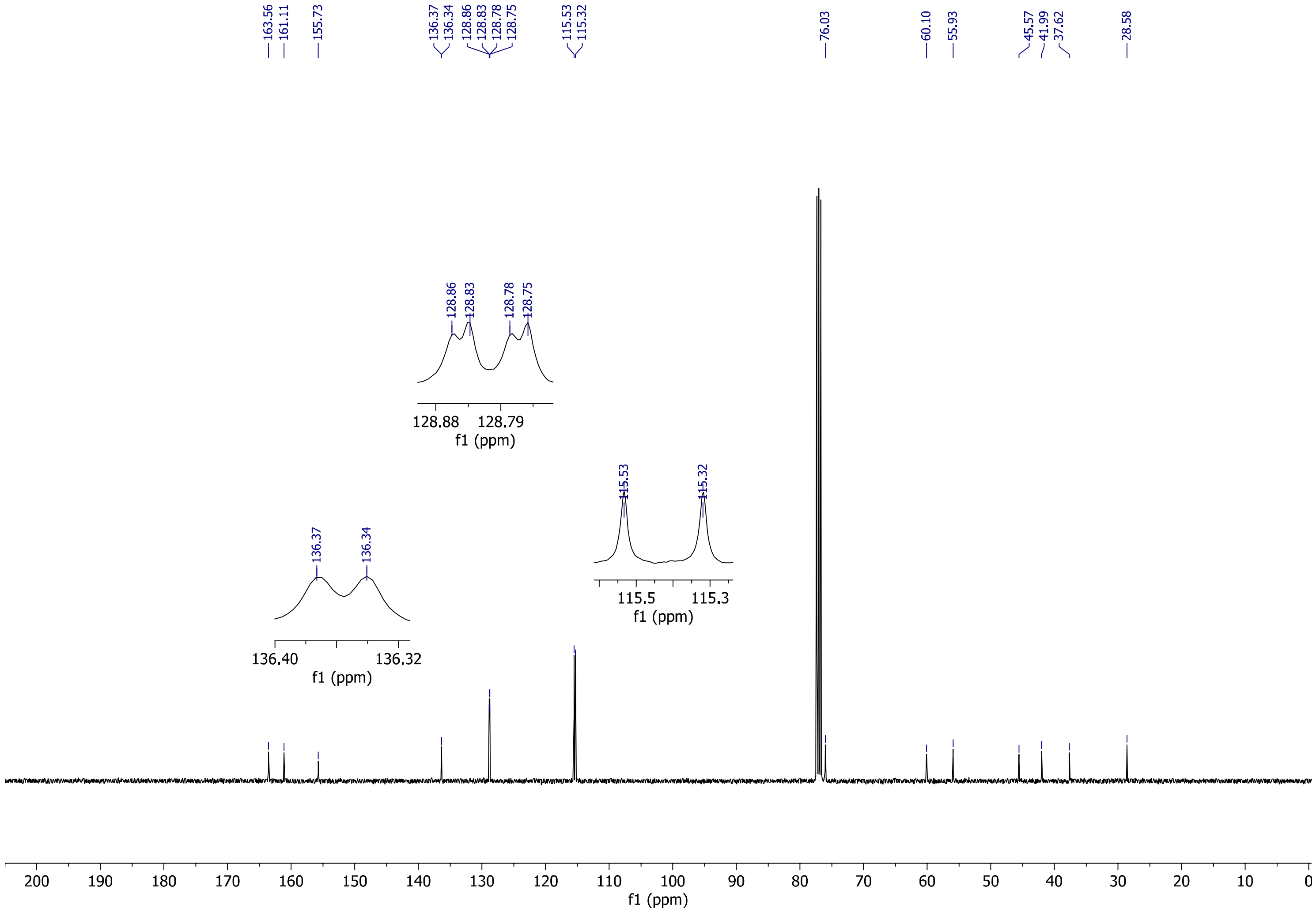

Figure S38. ^{13}C NMR spectrum of **11** (100 MHz, $CDCl_3$).

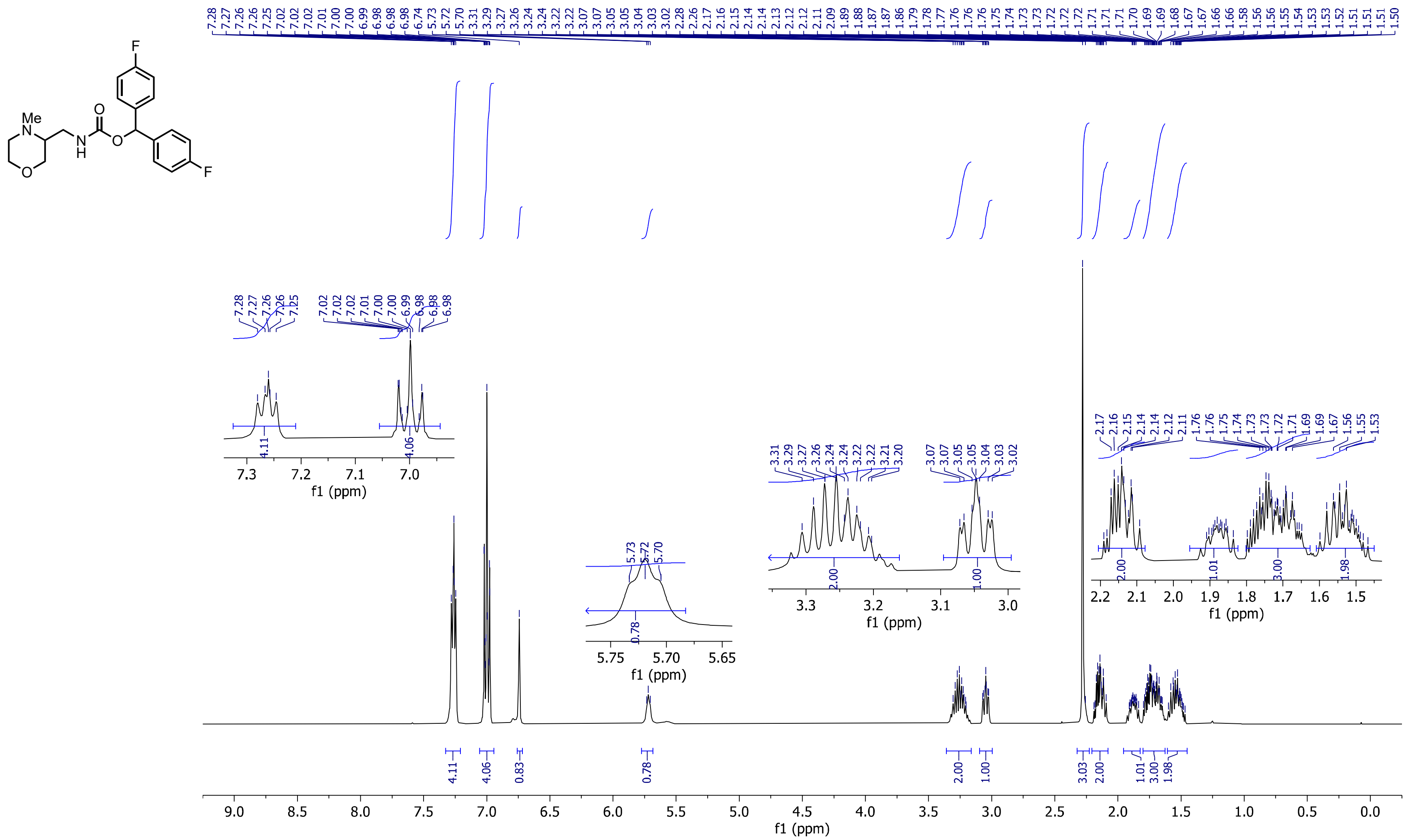

Figure S39. ^{1}H NMR spectrum of **12** (400 MHz, $CDCl_3$).

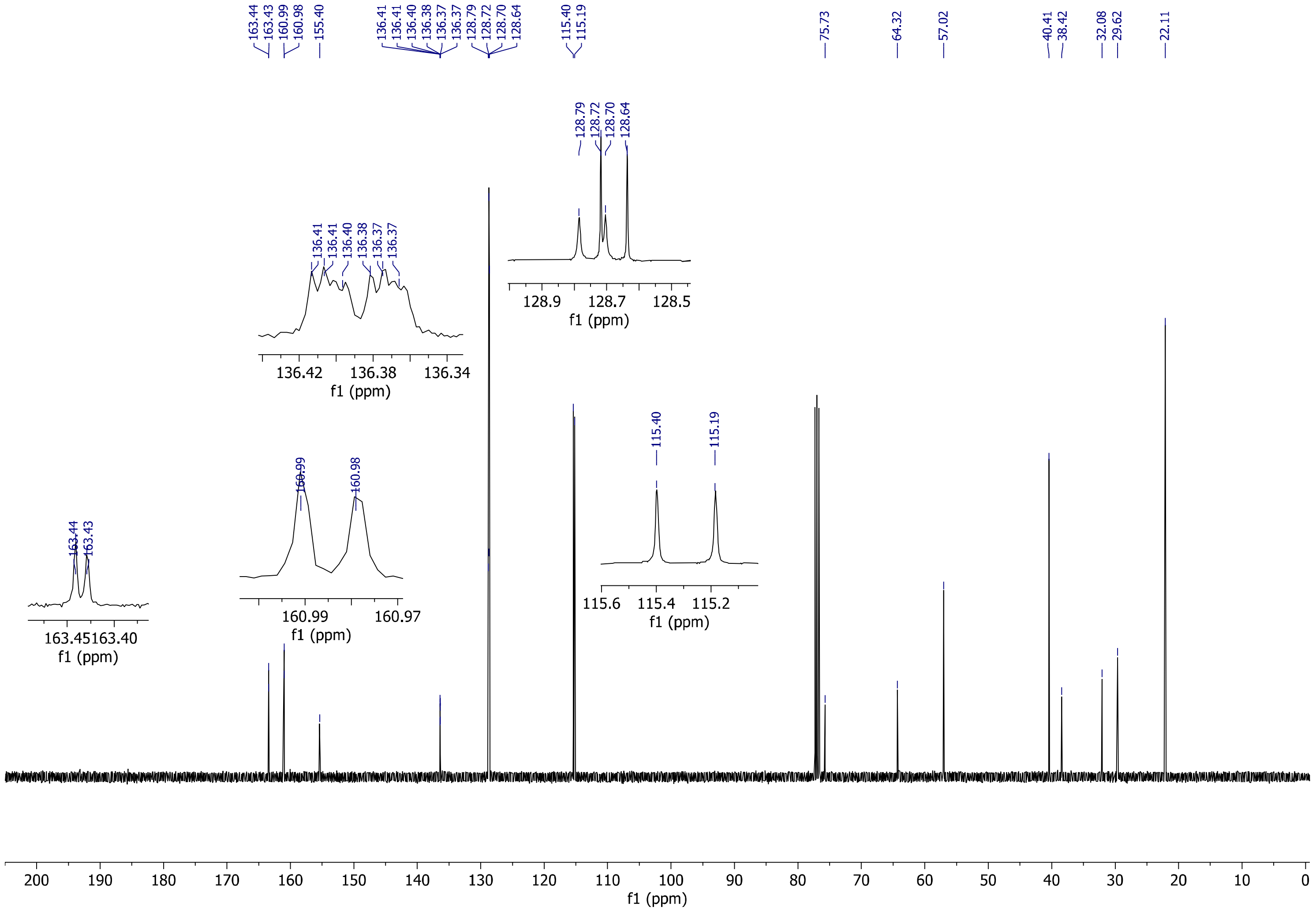

Figure S40. ^{13}C NMR spectrum of **12** (100 MHz, $CDCl_3$).
